# Efficacy and Safety of Cannabis Derivates and Their Synthetic Analogs. Overview of Systematic Reviews

**DOI:** 10.1002/ptr.70403

**Published:** 2026-06-23

**Authors:** Rafael Leite Pacheco, Daniela Fortunato Rêgo, Carolina de Oliveira Cruz Latorraca, Marco Aurelio de Carvalho Nascimento, André Bastos Daher, Rachel Riera

**Affiliations:** ^1^ Centro Universitário São Camilo, Medicine São Paulo Brazil; ^2^ Hospital Sírio‐Libanês, Núcleo de Avaliação de Tecnologias Em Saúde São Paulo Brazil; ^3^ Ministério dos Direitos Humanos e da Cidadania Brasília Distrito Federal Brazil; ^4^ Oswaldo Cruz Foundation (Fiocruz) Rio de Janeiro Brazil; ^5^ Plataforma de Pesquisa Clínica da Fiocruz, Vice Presidency of Research and Biological Collections (VPPCB), Oswaldo Cruz Foundation (Fiocruz) Rio de Janeiro Brazil; ^6^ Universidade Federal de São Paulo, Escola Paulista de Medicina São Paulo Brazil

## Abstract

The use of medicinal products derived from cannabis and its synthetic analogues has grown in recent years for various health conditions, which led to an increase in systematic reviews (SR) on the topic. The objective of this overview was to identify, synthesize, and critically appraise the evidence from SR on the benefits and harms of cannabis derivatives when used for therapeutic purposes in different health conditions. A comprehensive search was conducted to identify all relevant SR in Embase, Epistemonikos, MEDLINE, and Cochrane Database of Systematic Reviews. The inclusion criteria was SR assessing the effects of cannabis and its derivatives for any clinical condition that included only randomized controlled trials. A structured selection and extraction process was performed by two independent researchers. The methodological quality of the included SR was assessed using AMSTAR‐2. This overview included 102 SR, 68.6% of which were of critically low quality and 17.6% were of high quality. There is low to moderate certainty of evidence of benefits from these interventions for ulcerative colitis, chronic non‐cancer pain, Crohn's disease, multiple sclerosis, and post‐chemotherapy nausea and vomiting. The results point to a lack of benefits for sleep disorders, chronic cancer pain refractory to opioids, pain related to radiotherapy, and pain in people receiving palliative care. For other conditions, the certainty of the evidence was very low or not assessed. This overview opens a broad and complex field for the development of primary studies to evaluate the effects of cannabinoids as primary or adjunctive therapy for different health conditions and reinforces the importance of safety assessment. Decision‐makers and guideline developers can be guided by the results summarized in this overview. However, when making formal recommendations, it is essential to consider the quality of the SR and the certainty of the evidence identified for each outcome.

## 1 Introduction

The use of medicinal products derived from *cannabis* sp. and its synthetic analogues has grown in recent years in different countries and for various health conditions (Boehnke et al. 2024; Cremer‐Schaeffer and Langer 2024).

There has also been an exponential increase in clinical studies, although this growth does not necessarily result in robust confidence in the body of evidence for the health conditions analysed, which ultimately compromises the practical applicability of the clinical results observed (United States. National Institutes of Health 2025). This context stems from the fact that most clinical trials conducted are of low methodological quality, as they group signs and symptoms for different diseases in their analyses and present one result for clinically heterogeneous populations, for different doses, periods, and routes of drug administration (Riera et al. 2022).

Despite this scenario, there is evidence of the benefits of some cannabinoids for diseases where there are no or few therapeutic alternatives, which justifies the need for further research in this area (Riera et al. 2022).

Given the continuous increase in systematic reviews on the therapeutic effects derived from *cannabis* sp. and its synthetic analogues, it is essential to identify and critically evaluate the quality of these reviews in order to: (a) strengthen the body of evidence to support clinical practice, (b) inform decision‐making regarding the incorporation of new technologies into health systems, (c) guide the productive and technological development of new drugs, and (d) identify gaps and direct future research on the efficacy and safety of cannabis derivatives and their synthetic analogues for therapeutic use.

## 2 Objective

To identify, synthesize, and map the evidence from systematic reviews on the effects (benefits and harms) of *cannabis* sp. derivatives—including the whole plant, its extracts, and synthetic analogues—when used for therapeutic purposes in different health conditions.

## 3 Methods

This overview of systematic reviews was developed at the Centre for Teaching and Research in Evidence‐Based Health Care and Health Technology Assessment and Education (NEP‐SBEATS) at the Universidade Federal de São Paulo (Unifesp). The overview was conducted following the methodological guidelines of the Cochrane Handbook for Systematic Reviews of Interventions (Higgins et al. 2024), and this article was written according to the relevant items of the Preferred Reporting Items for Systematic Reviews and Meta‐Analyses (PRISMA 2020) (Page et al. 2021).

### 3.1 Elegibility Criteria

#### 3.1.1 Types of Reviews

All systematic reviews that included only randomized controlled trials as primary study designs were considered. Systematic review protocols, systematic reviews marked as “withdrawn” in the Cochrane Database of Systematic Reviews (CDSR), previous versions of the same review, and reviews published only as abstracts were not considered.

To be eligible, a study had to meet all of the following criteria: (i) the title and/or abstract explicitly stated that the study was a systematic review, (ii) there was a detailed report of a structured search strategy used in at least one database, and (iii) there was a systematic assessment of the quality of the included studies, along with a description of the critical appraisal strategy used. Rapid reviews, scoping reviews, other evidence syntheses, and health technology assessment reports were not included.

#### 3.1.2 Types of Participants

Children and/or adults with any clinical condition. Systematic reviews including healthy people with no health conditions were not considered.

#### 3.1.3 Types of Interventions

All forms derived from *cannabis* sp. were considered, including whole plant or extract, and their synthetic analogues, alone or in combination, in any regimen, dose, or route of administration, when used for therapeutic purposes. When a cannabis‐derived product was evaluated as add‐on to another treatment, only comparisons in which the comparator group received the same cointervention were considered. For example, “cannabis‐derived product plus standard care” was compared only with “placebo plus standard care”, not with standard care alone.

Any active treatment, placebo, waiting list (a group of participants scheduled to receive the intervention later), or no intervention was considered as a comparator.

Systematic reviews that compared only different presentations of *cannabis* sp., dosage, or regimen schedules were not considered.

### 3.2 Outcomes of Interest

- Primary outcomes

Clinical assessment specific to the health condition, using any validated tool or measurement.

Serious adverse events, as defined by the authors of the included systematic review.

- Secondary outcomes

Quality of life, using any validated tool.

Any adverse event, as defined by the authors of the included systematic review.

Findings for primary and secondary outcomes are reported jointly by clinical condition rather than separated by outcome type, given that definitions of primary and secondary outcomes varied across the included systematic reviews.

Eligibility criteria were established a priori in the study protocol and were intentionally kept broad regarding clinical conditions and outcome instruments. Because this overview was designed to map all systematic reviews on cannabis derivatives and synthetic analogues, no closed list of conditions or assessment tools was pre‐specified. The clinical conditions and outcome instruments effectively assessed in the included reviews are detailed in Supporting Informations S3 and S5.

### 3.3 Search Strategy

A comprehensive and sensitive search was conducted to identify all relevant systematic reviews. The search strategies considered the controlled vocabulary of each database, the terms listed in the hierarchical tree, and their synonyms. Where possible, validated systematic review filters were also used.

The following electronic databases were considered:

- Cochrane Database of Systematic Reviews, via Cochrane Library.
- Epistemonikos.
- Excerpta Medica database (Embase, via Elsevier).
- Medical Literature Analysis and Retrieval System Online (MEDLINE, via PubMed).

The search strategies developed for each database are presented in Supporting Information S1. Additional manual searches were conducted in the reference lists of the included reviews and in other clinical practice guidelines relevant to the area.

### 3.4 Selection Process

The selection process was conducted in two stages, independently by two reviewers (RLP and COCL), and any conflicts were solved by a third researcher (RR). The selection process occurred using the Rayyan platform (Ouzzani et al. 2016).

In the first stage, the reviewers identified potentially eligible studies by screening the titles and abstracts of all references retrieved by the search strategies. References that clearly did not meet the eligibility criteria were eliminated at this stage.

The second stage consisted of screening the references marked as potentially eligible in the first stage to confirm eligibility or exclusion. The references and reasons for exclusions in the second stage are presented in Supporting Information S2.

### 3.5 Data Extraction Process

A standardized extraction spreadsheet was used to extract relevant data from the included studies. The data extraction process was conducted independently by two reviewers (RLP and COCL), and discrepancies were resolved by consulting a third author (RR). A pilot extraction considering 10 included reviews was performed to test and refine the extraction spreadsheet.

The following data were considered for extraction:

- Main methodological characteristics of the systematic review.
- Number of randomized clinical trials included.
- Number of participants included.
- Type and description of cannabinoids considered.
- Results of outcomes of interest.
- Assessment of the certainty of evidence using the GRADE approach (Guyatt et al. 2008), when available in the included reviews.
- Conflicts of interest and funding sources.

### 3.6 Methodological Quality

The methodological quality of the included systematic reviews was assessed using the Assessment of Methodological Quality of Systematic Reviews (AMSTAR‐2) tool (Shea et al. 2017). The AMSTAR‐2 tool consists of 16 domains that can be judged as fully adequate (“yes”), partially adequate (“partially yes”), inadequate (“no”), or not applicable (NA). Seven of these domains are considered critical (1, 4, 7, 9, 11, 13, and 15).

The overall quality of the reviews was rated using the AMSTAR‐2 tool as follows: critically low (more than one critical flaw), low (one critical flaw), moderate (more than one non‐critical flaw), and high (no or one non‐critical flaw).

The assessments were performed independently by two reviewers (RLP and COCL), with a third researcher solving any disagreements (RR). All domain‐level consensus judgments are presented for all included reviews, as well as the percentage of adequate items for each review.

The proportion of studies that met the criteria for each item of AMSTAR‐2 tool was calculated as a percentage, where the numerator was the number of items with a “YES” response and the denominator was the total number of items assessable by the tool, i.e., excluding those classified as “not applicable.”

### 3.7 Certainty of Evidence

The assessment of the certainty of the evidence (GRADE) presented in the included reviews was extracted and presented narratively together with the related outcome results. When the certainty of the evidence was presented, the GRADE recommendations for the narrative of the results were adopted; when there was no assessment of certainty, the narrative for presenting the results was restricted to the statistical difference (Guyatt et al. 2008).

The availability of evidence certainty assessment using the GRADE approach is an important aspect of supporting decision‐making. For each outcome estimate, GRADE considers five domains of assessment (risk of bias, imprecision, inconsistency, indirectness, and publication bias) to estimate how reliable the numerical estimate is GRADE facilitates the interpretation of results and allows conclusions and recommendations to be drawn up in a standardized manner. GRADE has a reporting terminology that considers the certainty of evidence, as in the examples:

- High certainty: “the intervention *reduces* the risk of the outcome”.
- Moderate certainty: “the intervention *probably* reduces the risk of the outcome”.
- Low certainty: “the intervention *may* reduce the risk of the outcome”.
- Very low certainty: “the effects of the intervention are *very uncertain*.”

### 3.8 Results Synthesis

Descriptive statistics were used to summarize the characteristics of the included studies. The included systematic reviews were grouped by health condition to synthesize the extracted outcome data. When two or more systematic reviews on the same health condition were included, the systematic review with the best methodological quality according to AMSTAR‐2 and with the most recent search date was considered to compose the synthesis of results (Shea et al. 2017). Exceptions were discussed among the reviewers, and deviations from this rule were noted in the corresponding sections.

The statistical results of the outcome assessment were extracted as reported by the included systematic reviews. Effect size estimates (risk ratio, odds ratio, and mean difference) were reported along with the respective 95% confidence interval (when available). Data management and tabulation were performed using Stata version 18.

## 4 Results

### 4.1 Search Results

The searches retrieved 4562 references. After excluding 875 duplicates, 3687 references had their titles and abstracts evaluated. After the first phase of the selection process, 189 studies were considered potentially eligible and were evaluated in full text in the second phase of selection to confirm eligibility. At the end, 87 studies were excluded and are presented in the Supporting Information S2 with the reasons for exclusion. Thus, 102 systematic reviews were included in this overview (Alderman et al. 2022; Amato et al. 2017; Aminilari et al. 2022; Andreae et al. 2015; Aviram and Samuelly‐Leichtag 2017; Bajtel et al. 2022; Barakji et al. 2023; Belgers et al. 2021; Bilbao and Spanagel 2022; Boland et al. 2020; Bonaccorso et al. 2019; Boychuk et al. 2015; Campbell et al. 2001; Cárdenas et al. 2025; Charernboon et al. 2021; Chesney et al. 2020; Chhabra et al. 2024; Choi et al. 2024; Chow et al. 2020, 2025; Coelho et al. 2024; Curtis et al. 2009; Deshpande et al. 2015; Fazlollahi et al. 2023; Filippini et al. 2022; Fisher et al. 2021; Fitzcharles, Baerwald, et al. 2016; Fitzcharles, Ste‐Marie, et al. 2016; Gazendam et al. 2020; Gedin et al. 2022; Gloss and Vickrey 2014; Guerrini et al. 2024; Hammond et al. 2021; Hassan 2018; Häuser et al. 2019, 2023; Iskedjian et al. 2007; Jeddi et al. 2024; Johal et al. 2020; Kafil et al. 2018a, 2018b; Kang et al. 2025; Kleiner et al. 2023; Köck et al. 2024; Kuharic et al. 2021; Kumar et al. 2024; Lakhan and Rowland 2009; Lattanzi, Brigo, Cagnetti, et al. 2018; Lattanzi, Brigo, Trinka, et al. 2018; Lattanzi et al. 2020; Lim et al. 2017; Linley et al. 2022; Longo et al. 2021; Lopes Júnior et al. 2023; Lutge et al. 2013; Lynch and Ware 2015; Machado Rocha et al. 2008; Martin et al. 2023; Martín‐Sánchez et al. 2009; McKee et al. 2021; McLoughlin et al. 2014; McParland et al. 2023; Meng et al. 2017; Mücke, Phillips, et al. 2018; Mücke, Weier, et al. 2018; Oikonomou and Jost 2022; Oliveira et al. 2025; Paunescu et al. 2020; Petzke et al. 2016; Quintero et al. 2022; Rabgay et al. 2020; Razmovski‐Naumovski et al. 2022; Reechaye et al. 2024; Rovare et al. 2017; Ruthirakuhan et al. 2019; Sainsbury et al. 2021; Seneca 2014; Smith et al. 2015; Souza et al. 2022; Spanagel and Bilbao 2021; Stevens and Higgins 2017; Talwar et al. 2023; Tateo 2017; Torres and Farré 2015; Torres‐Moreno et al. 2018; Tramèr et al. 2001; Treves et al. 2021; Tsai et al. 2022; Vaid et al. 2025; van den Elsen et al. 2014; Velayudhan et al. 2024; Volz et al. 2016; Votrubec et al. 2022; Vuilleumier et al. 2022; Walitt et al. 2016; Wang et al. 2019, 2021, 2024; Werneck et al. 2018; Whiting et al. 2015; Wong et al. 2020; Zhang et al. 2022).

For one of the reviews included on the effects of cannabinoids on Alzheimer's disease (Ruthirakuhan et al. 2019), it was not possible to obtain the full text of the publication; thus, its main characteristics were presented, but it was not possible to assess its methodological quality, and its results were not considered for presentation.

The entire process of selecting studies for inclusion is presented in detail in the PRISMA flowchart in Figure 1.

**FIGURE 1:**
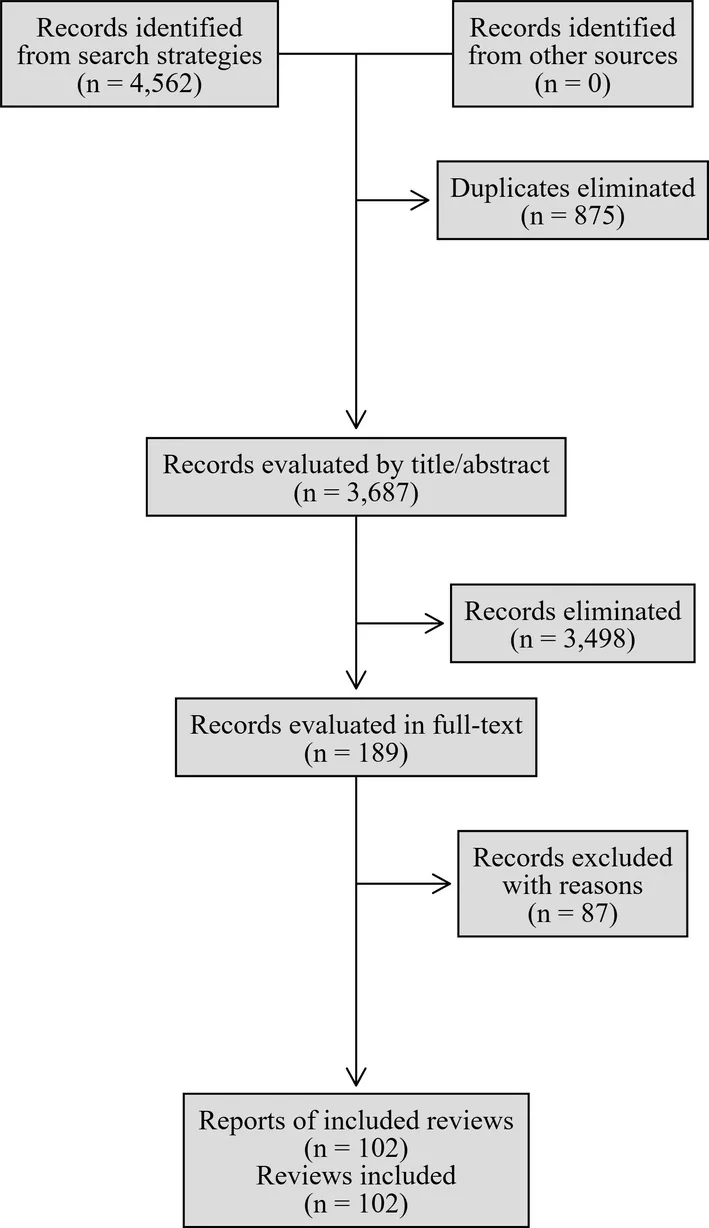
Flowchart of the selection process.

The reviews included were published between 2001 (Campbell et al. 2001; Tramèr et al. 2001) and 2025 (Cárdenas et al. 2025; Chow et al. 2025; Kang et al. 2025; Oliveira et al. 2025; Vaid et al. 2025). The systematic reviews included between 152 clinical trials (Bilbao and Spanagel 2022) and one clinical trial (Quintero et al. 2022), with an average of 16 clinical trials per review.

Among the 102 reviews included, 26.5% (27/102) assessed cannabis derivatives for the management of more than one different symptom or clinical condition, and 9.8% (10/102) included clinical trials that analysed any disease or clinical condition (Bajtel et al. 2022; Bilbao and Spanagel 2022; Chesney et al. 2020; Chhabra et al. 2024; Souza et al. 2022; Spanagel and Bilbao 2021; Treves et al. 2021; van den Elsen et al. 2014; Velayudhan et al. 2024; Whiting et al. 2015).

Thirty‐seven reviews (36.2%) assessed clinical trials on pain in general or of different origins, such as neuropathic, chronic, post‐operative, oncological, non‐oncological, rheumatological, acute, and orofacial pain. Twenty‐four (23.5%) reviews assessed neurological syndromes. Cancer‐related symptoms, such as pain, cachexia, or nausea and vomiting, were assessed by 14.7% of the reviews.

Psychiatric disorders were the population of interest in 10.8% of the included reviews, and gastrointestinal symptoms and diseases (other than cancer‐related nausea and vomiting) were also addressed by 6.0% of the reviews, including Crohn's disease, inflammatory bowel disease, ulcerative colitis, and gastrointestinal diseases in general.

Fifteen reviews (14.7%) assessed cannabidiol alone, three (2.9%) assessed different cannabinoids, one assessed nabiximols exclusively (Kleiner et al. 2023), and one assessed the combination of cannabidiol/THC phytocannabinoids (Lakhan and Rowland 2009).

Regarding comparators, 63 (61.8%) systematic reviews considered placebo and other active interventions, one review considered placebo or conventional analgesics (Cárdenas et al. 2025), one review considered fenfluramine (Linley et al. 2022), and one review considered opioids (Jeddi et al. 2024).

The main characteristics of the systematic reviews included are presented in detail in the Supporting Information S3.

### 4.2 Methodological Quality

The methodological quality and judgement for each AMSTAR‐2 item of the included systematic reviews is presented in Supporting Information S4. The average frequency of compliance with AMSTAR‐2 items among the reviews was 55%, and one review achieved 100% compliance with the items (Häuser et al. 2023), while four complied with only 1 item of the 16 items in the tool (Hassan 2018; Longo et al. 2021; Oikonomou and Jost 2022; Seneca 2014). Seventy reviews (68.6%) were considered as critically low quality and 18 (17.6%) of high quality. Figure 2 presents the percentage of adequacy to AMSTAR‐2 items from the included reviews.

**FIGURE 2:**
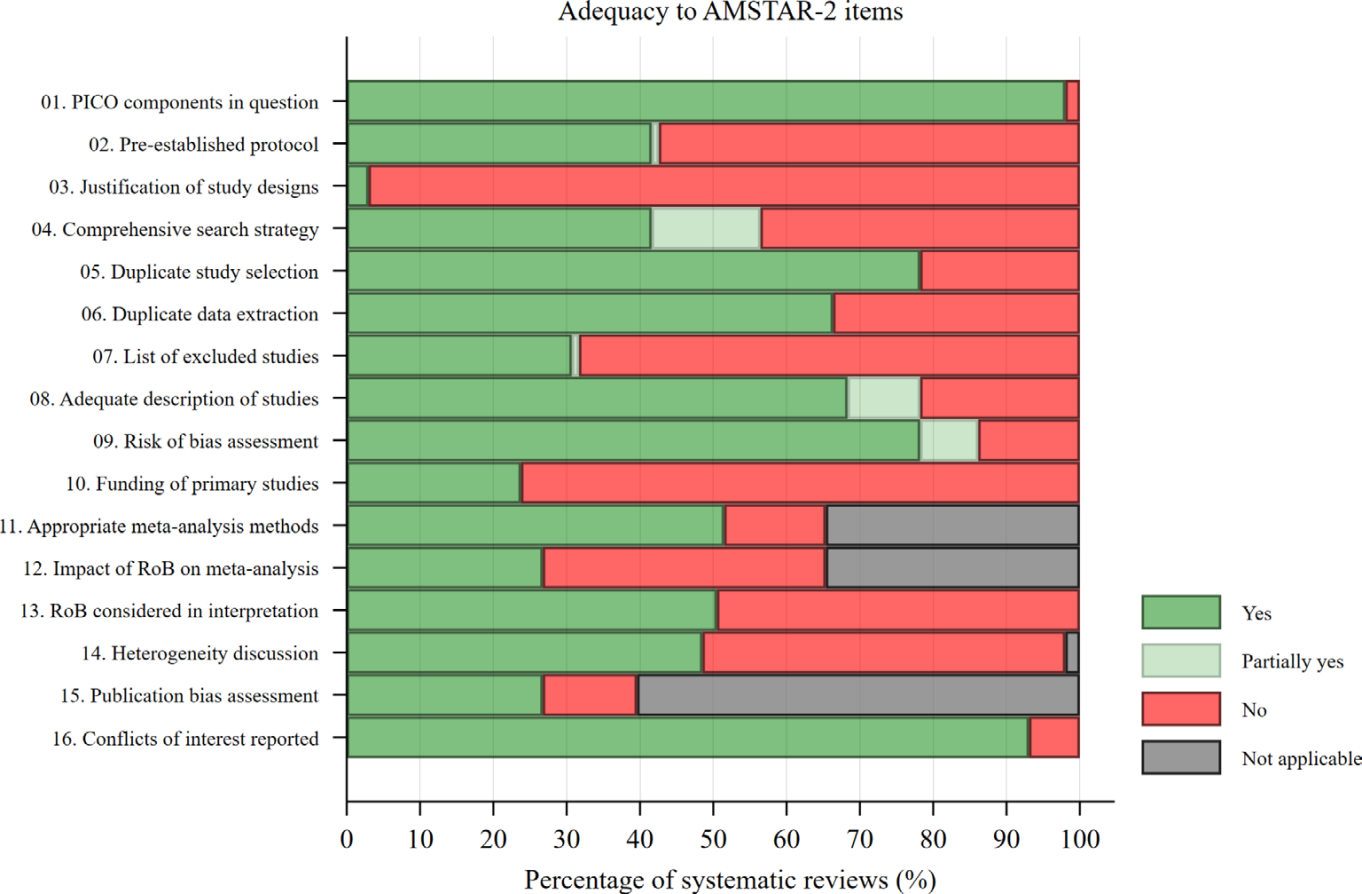
Adequacy of the included systematic reviews to AMSTAR‐2 items. AMSTAR‐2 items: (1) Did the research questions and inclusion criteria for the review include the components of PICO? (2) Did the report of the review contain an explicit statement that the review methods were established before the conduct of the review and did the report justify any significant deviations from the protocol? (3) Did the review authors explain their selection of the study designs for inclusion in the review? (4) Did the review authors use a comprehensive literature search strategy? (5) Did the review authors perform study selection in duplicate? (6) Did the review authors perform data extraction in duplicate? (7) Did the review authors provide a list of excluded studies and justify the exclusions? (8) Did the review authors describe the included studies in adequate detail? (9) Did the review authors use a satisfactory technique for assessing the risk of bias (RoB) in individual studies that were included in the review? (10) Did the review authors report on the sources of funding for the studies included in the review? (11) If meta‐analysis was performed, did the review authors use appropriate methods for statistical combination of results? (12) If meta‐analysis was performed, did the review authors assess the potential impact of RoB in individual studies on the results of the meta‐analysis or other evidence synthesis? (13) Did the review authors account for RoB in primary studies when interpreting/discussing the results of the review? (14) Did the review authors provide a satisfactory explanation for, and discussion of, any heterogeneity observed in the results of the review? (15) If they performed quantitative synthesis did the review authors carry out an adequate investigation of publication bias (small study bias) and discuss its likely impact on the results of the review? (16) Did the review authors report any potential sources of conflict of interest, including any funding they received for conducting the review?

### 4.3 Synthesis of the Results and Certainty of Evidence

The clinical conditions, comparisons, and efficacy and safety outcomes assessed for each health condition, considering the systematic reviews with the best methodological quality (AMSTAR‐2) and/or most recent searches, are presented in Supporting Information S5.

GRADE was used by the included systematic reviews to assess the certainty of evidence on efficacy in 59% (23/39) of the identified health conditions. GRADE was not used by any systematic review that focused exclusively on safety. No high‐certainty evidence was identified for the conditions assessed. Table 1 depicts cannabis‐derived product and dosing regimens for the outcomes included in the outcome synthesis.

**TABLE 1:** Cannabis‐derived products and dosing regimens for the conditions with at least one GRADE‐assessed outcome.

Condition	Product evaluated	Dose	Route	Duration
Acute pain	Cannabinoids (different dosages and presentations) vs. placebo	Variable	Oral	Not specified
Anxiety disorder	CBD (99.9% pure diluted in corn oil) vs. placebo	600 mg, single dose	Oral	2 h
Chronic cancer pain refractory to opioids	Nabiximols (Sativex) vs. placebo	Average 6.3 to 11 doses/day (THC 2.7 + CBD 2.5 mg/dose)	Oromucosal spray	Not specified
Chronic cancer pain, palliative care	CBD oil vs. placebo	50 to 600 mg/day	Oral	4 weeks
Chronic cancer pain related to radiotherapy	Nabilone vs. placebo	Up to 2 mg/day	Oral	8 weeks
Chronic neuropathic cancer pain	Nabilone vs. placebo	Not specified	Oral	8 weeks
Chronic non‐cancer pain	Cannabinoids (different dosages and presentations) vs. placebo	Variable	Oral	1 to 4 weeks
Crohn's disease	Cannabis cigarette (THC) vs. cannabis cigarette without THC	115 mg THC twice daily	Inhaled	8 weeks
Crohn's disease	Cannabis oil (CBD 5%) vs. placebo	2 mL twice daily	Sublingual	8 weeks
Crohn's disease	Cannabis oil (CBD 15% + THC 4%) vs. placebo	Not specified	Sublingual	8 weeks
Dementia	Nabilone vs. placebo	0.5 to 2 mg/day	Oral	6 weeks
Dementia	Natural THC (delta THC 98%) or dronabinol or nabilone vs. placebo	Variable	Oral	3 to 14 weeks
Fibromyalgia	Nabilone vs. placebo	0.5 to 1 mg twice daily	Oral	4 weeks
Fibromyalgia	Nabilone vs. amitriptyline	0.5 or 1 mg/day vs. 10 or 20 mg/day	Oral	2 weeks
Irritable bowel syndrome	Dronabinol 2.5 mg vs. dronabinol 5 mg vs. placebo	Single dose	Oral	Single dose
Multiple sclerosis	Cannabinoids (different dosages and presentations) vs. placebo	Variable	Oral; oromucosal	4–48 weeks
Parkinson's disease	Nabilone vs. placebo	2 mg/day	Oral	4 weeks
Post‐chemotherapy nausea and vomiting	Cannabinoids (different dosages and presentations) vs. placebo	Variable	Oral	Not specified
Post‐chemotherapy nausea and vomiting	Dronabinol vs. placebo	50 mg/day/m^2^ (5 doses)	Oral	1 day
Post‐chemotherapy nausea and vomiting	Cannabinoids (different dosages and presentations) vs. prochlorperazine (different dosages and presentations)	Variable	Oral	Not specified
Sleep disorders — any disorder	Dronabinol vs. placebo	Variable	Oral	Variable
Ulcerative colitis	CBD + THC (up to 4.7%) oral capsules vs. placebo	100–500 mg/day	Oral	10 weeks
Ulcerative colitis	Cannabis cigarette vs. placebo	0.5 mg twice daily (up to 23 mg THC/day)	Inhaled	8 weeks

*Note:* The table includes only conditions and comparisons for which at least one outcome was assessed using the GRADE approach by the systematic review used to compose the synthesis (see Table 2).

Abbreviations: CBD, cannabidiol; THC, Δ‐9‐tetrahydrocannabinol.

Table 2 presents the results, by condition, of the outcomes for which the certainty of the evidence was assessed (GRADE), considering the quality of the systematic review that mapped these results.

**TABLE 2:** Results, by condition, of the outcomes for which the certainty of the evidence was assessed (GRADE), considering the quality of the systematic review that mapped these results.

Condition	Comparison	Outcome results	Certainty of evidence (GRADE)	Quality of the systematic review (AMSTAR‐2)
Acute pain	Cannabinoids (different dosages and presentations) versus placebo	Pain (ENA): the effects of nabilone are uncertain (DM 0.52; 95% CI −0.40 to 1.43; *p* = 0.19; *I* ^2^ = 90%; 5 RCTs, 530 participants)	Very low	Low
Anxiety disorder	CBD 600 mg (99.9% pure diluted in corn oil) versus placebo, 2 h	Anxiety level (Visual Analogue Mood Scale—VAMS, score 0–100): lower with cannabidiol (MD −16.52; *p* = 0.1; 1 EC, 24 participants)	Very low	Moderate
Chronic cancer pain refractory to opioids	Nabiximols (Sativex spray, THC 2.7 mg + CBD 2.5 mg, average of 6.3 to 11 doses/day) versus placebo	Pain (improvement or much improvement in PGIC): cannabinoids probably do not improve PGIC (RR 0.06, 95% CI 0.01 to 0.12; 4 EC, 996 participants)	Moderate	High
		Maintained average daily opioid dose: cannabinoids may not alter the daily dose of opioids (SMD 0.08, 95% CI −0.10 to 0.27; 3 EC, 970 participants)	Low	High
		Serious adverse events: cannabinoids probably do not alter the risk of serious adverse events (RR 0.02, 95% CI −0.03 to 0.07; 4 EC, 1330 participants)	Moderate	High
		Dropout due to adverse events: cannabinoids probably do not alter the risk of dropout (RR: 0.04; 95% CI 0.0 to 0.08; 4 EC, 1332 participants)	Moderate	High
Chronic cancer pain, people receiving palliative care	CBD 50–600 mg/day, oral oil versus placebo, 4 weeks	Pain (≥ 30% improvement): 26/70 participants with CBD versus 29/72 with placebo; *p* = not reported; 1 RCT, 142 participants	Low	High
Chronic cancer pain related to radiotherapy	Nabilone up to 2 mg/day versus placebo, 8 weeks	Pain (≥ 30% improvement): 7/14 participants with nabiximols versus 7/19 with placebo; *p* = 0.45; 1 RCT, 38 participants	Low	High
		Pain (≥ 50% improvement): 5/14 participants with nabiximols versus 5/19 with placebo; *p* = 0.56; 1 RCT, 38 participants	Low	High
Chronic neuropathic cancer pain	Nabilone versus placebo, 8 weeks	Pain (improvement ≥ 30%): 5/18 participants with nabilone versus 3/18 with placebo; *p* = 0.6; 1 RCT, 36 participants	Low	High
		Pain (≥ 50% improvement): 2/18 participants with nabilone versus 3/18 with placebo; *p* = 0.47; 1 RCT, 36 participants	Low	High
		Adverse events affecting the central nervous system: may be more frequent with nabiximols (9/18 participants with nabilone versus 0/18 with placebo; *p* = 0.02; 1 EC, 36 participants)	Low	High
		Central nervous system adverse events: 3/18 participants with nabilone versus 0/18 with placebo; *p* = 0.23; 1 EC, 36 participants	Low	High
Chronic non‐cancer pain	Cannabinoids (different dosages and presentations) versus placebo, 1 to 4 weeks	Pain (VAS): cannabinoids probably reduce VAS scores, but this reduction does not reach the minimum clinically relevant difference (SMD −0.63; 95% CI −0.96 to −0.29; 23 EC, 2580 participants).	Moderate	High
Crohn's disease	Cannabis cigarette (115 mg THC) twice daily versus cannabis cigarette without THC, 8 weeks	Clinical remission (CDAI score < 150): the effects of smoking cannabis containing THC are very uncertain (RR 4.55, 95% CI 0.63 to 32.56; 1 EC; 21 participants)	Very low	High
		Clinical response (reduction in CDAI ≥ 100): the effects of smoking cannabis containing THC are very uncertain (RR 2.27, 95% CI 1.04 to 4.97; 1 EC; 21 participants)	Very low	High
		Adverse events: the effects of smoking cannabis containing THC are very uncertain (RR 4.09; 95% CI 1.15 to 14.57; 1 EC; 21 participants)	Very low	High
	Cannabis oil (CBD 5%, sublingual use) 2 mL twice daily versus placebo for 8 weeks	Clinical remission (CDAI score ≤ 150): the effects of cannabis oil are very uncertain (RR 1.20, 95% CI 0.36 to 3.97; 1 EC; 19 participants)	Very low	High
		Serious adverse events: the effects of cannabis oil are very uncertain (RR 0.90, 95% CI 0.07 to 12.38; 1 EC; 19 participants)	Very low	High
	Cannabis oil (CBD 15% and THC 4%, sublingual use) versus placebo for 8 weeks	Quality of life (SF‐36): cannabis oil may increase SF‐36 scores (MD 16.4, 95% CI 5.72 to 27.08; 1 EC, 39 participants)	Low	High
Dementia	Nabilone 0.5 mg/day to 2 mg/day versus placebo, 6 weeks	Cognitive function (MiniMental): no clinically relevant difference between groups, but this result is very uncertain (MD 1.10, 95% CI 0.10 to 2.10; 1 cross‐over EC, 38 participants)	Very low	High
		Psychological/behavioural symptoms (NPI‐NH scale): may be better with nabilone (MD −4.6, 95% CI −7.5 to −1.7; 1 cross‐over EC; 38 participants)	Low	High
	Natural THC (delta THC 98%) or dronabinol or nabilone versus placebo, 3 to 14 weeks	Psychological/behavioural symptoms (NPI‐NH or NPI, score from 0 to 144; the higher the score, the more symptoms): DM −1.97; 95% CI −3.87 to 0.07; 3 RCTs, 111 participants	Low	High
		Agitated or aggressive behaviours (CMAI, score from 29 to 203; the higher the score, the more symptoms): MD –2.35; 95% CI –4.10 to −0.60; 3 EC, 111 participants	Low	High
		Overall adverse events: OR 0.70; 95% CI 0.11 to 4.38; 1 EC, 50 participants	Low	High
Fibromyalgia	Nabilone 0.5 mg to 1 mg twice daily versus placebo, 4 weeks	Pain (EVA, score 0 to 10): 4.8 ± 2.2 with nabilone versus 5.7 ± 1.8 with placebo; *p* = 0.02; 1 EC, 20 participants	Very low	Low
		Fatigue (FIQ subscale): no difference between intervention groups (numerical results not reported; 1 RCT, 20 participants)	Very low	Low
		Anxiety (FIQ subscale): 4.3 ± 1.8 with nabilone versus 4.9 ± 2.2 with placebo; *p* < 0.01; 1 EC, 20 participants	Very low	Low
		Depression (FIQ subscale): no difference between intervention groups (numerical results not reported; 1 RCT, 20 participants)	Very low	Low
		Quality of life (total FIQ): 54 ± 22.3 with nabilone versus 64 ± 13.40 with placebo; *p* < 0.01; 1 EC, 20 participants	Very low	Low
		Withdrawal due to adverse events: 15% in the nabilone group versus 0% in the placebo group (1 RCT, 20 participants)	Very low	Low
		Adverse events (nabilone versus placebo): drowsiness 47% versus 6%; sialorrhea 33% versus 6%; vertigo 27% versus 0%; ataxia 20% versus 6%; confusion 13% versus 6%; loss of concentration 13% versus 6% (1 cross‐over EC, 29 participants)	Very low	Low
	Nabilone 0.5 mg or 1 mg/day versus amitriptyline 10 or 20 mg/day, 2 weeks	Pain (McGill Pain Questionnaire): effects uncertain (numerical results not reported; 1 cross‐over RCT, 29 participants)	Very low	Low
		Sleep (Insomnia Severity Index/Leeds Sleep Evaluation Questionnaire): effects are uncertain (±10.8 with nabilone versus 13 ± 10.81 with amitriptyline; cross‐over RCT, 29 participants)	Very low	Low
		Quality of life (total FIQ): effects are uncertain (numerical results not reported; 1 cross‐over RCT, 29 participants)	Very low	Low
		Discontinuation due to adverse events: effects are uncertain (3% in the nabilone group versus 0% in the placebo group; 1 cross‐over RCT, 29 participants)	Very low	Low
		Adverse events (nabilone versus amitriptyline): effects are uncertain (dizziness 32% versus 13%; nausea 29% versus 3%; 1 cross‐over EC, 29 participants)	Very low	Low
Irritable bowel syndrome	Dronabinol 2.5 mg versus dronabinol 5 mg versus placebo, single dose	Symptoms of the disease: the effects are uncertain (SMD −0.53, 95% CI −1.06 to 0.00; 1 RCT, 75 participants)	Very low	Critically low
Multiple sclerosis	Cannabinoids (different dosages and presentations) versus placebo, 4 to 48 weeks	Spasticity (frequency of participants reporting ≥ 30% reduction in spasticity, NNT): cannabinoids probably increase the frequency of participants with reduced spasticity (OR 2.51; 95% CI 1.56 to 4.04; *p* = 0.0002; 5 RCTs, 1143 participants)	Moderate	High
		Pain (frequency of participants reporting ≥ 50% reduction in pain, NNT): effects are uncertain (OR 4.23, 95% CI 1.11 to 16.17; 1 RCT, 48 participants)	Very low	High
		Quality of life: cannabinoids may have little or no effect on quality of life (SMD −0.08, 95% CI −0.17 to 0.02; 8 EC, 1942 participants; *I* ^2^ = 0%)	Low	High
		Perception of overall improvement: cannabinoids are likely to increase the number of people reporting a “very large” or “large” improvement in health status (OR 1.80, 95% CI 1.37 to 2.36; 8 EC, 1215 participants; *I* ^2^ = 0%)	Moderate	High
		Total adverse events: may be more frequent with cannabinoids (OR 1.38, 95% CI 0.96 to 1.99; 20 EC, 3124 participants; *I* ^2^ = 0%)	Low	High
		Adverse nervous system events: may be more frequent with cannabinoids (OR 2.61, 95% CI 1.53 to 4.44; 7 EC, 1154 participants; *I* ^2^ = 63%)	Low	High
		Psychiatric disorders: may be more frequent with cannabinoids (OR 1.94, 95% CI 1.31 to 2.88; 6 EC, 1122 participants; *I* ^2^ = 0%)	Low	High
		Treatment discontinuation due to adverse events: may be more frequent with cannabinoids (OR 2.41; 95% CI 1.51 to 3.84; 21 EC, 3110 participants; *I* ^2^ = 17%)	Low	High
Parkinson's disease	Nabilone 2 mg/day versus placebo, 4 weeks	Overall motor/non‐motor assessment (MDS‐UPDRS‐I): the effects of nabilone are uncertain (MD 1.63, 95% CI 0.09 to 3.18; *p* = 0.03; 1 RCT, 38 participants)	Very low	Critically low
		Anxiety (HADS‐A): the effects of nabilone are uncertain (MD −0.08, 95% CI −0.72 to 0.56; 1 EC, 38 participants)	Very low	Critically low
		Pain (EVA): the effects of nabilone are uncertain (MD –4.42, 95% CI –0.02 to 11.17; *p* = 0.96; 1 RCT, 38 participants)	Very low	Critically low
		Cognition (MoCA): the effects of nabilone are uncertain (MD –0.74; 95% CI –1.81 to 0.46; *p* = 0.053; 1 EC, 38 participants)	Very low	Critically low
		Fatigue (FSS): the effects of nabilone are uncertain (MD 4.00, 95% CI −3.53 to 11.453; *p* = 0.36; 1 EC, 38 participants)	Very low	Critically low
		Padrão do sono (ESS): os efeitos da nabilona são incertos (MD −0.05; IC 95% −1.5 a 1.45; *p* = 0.91; 1 EC, 38 participantes)	Very low	Critically low
		Sleep pattern (ESS): the effects of nabilone are uncertain (MD −0.05, 95% CI −1.5 to 1.45; *p* = 0.91; 1 EC, 38 participants)	Very low	Critically low
		Total adverse events: the effects of nabilone are uncertain (32% in the nabilone group versus 42% in the placebo group; RR 0.76; 95% CI not reported; 1 RCT, 38 participants)	Very low	Critically low
Post‐chemotherapy nausea and vomiting	Cannabinoids (different dosages and presentations) versus placebo	Absence of nausea and vomiting: cannabinoids probably increase the proportion of people without nausea and vomiting (RR 2.9, 95% CI 1.8 to 4.7; 3 EC, 288 participants)	Moderate	High
		Absence of vomiting: cannabinoids may increase the proportion of people without vomiting (RR 5.7; 95% CI 2.6 to 12.6; 3 EC, 168 participants)	Low	High
		Study dropout due to adverse events: the effects of cannabinoids on the risk of dropout are uncertain (RR 6.9, 95% CI 1.96 to 24; 2 EC, 276 participants)	Very low	High
	Dronabinol 50 mg/day/m^2^ asc versus placebo, 1 day (divided into 5 doses)	Absence of nausea: no difference between groups (RR 2.0, 95% CI 0.2 to 21; 2 EC, 96 participants)	Low	High
	Cannabinoids (different dosages and presentations) versus prochlorperazine (different dosages and presentations)	Absence of nausea: cannabinoids may increase the proportion of people without nausea (RR 1.46; 95% CI 0.67 to 3.15; *I* ^2^ = 58%; 5 EC, 258 participants)	Low	High
		Absence of vomiting: cannabinoids probably increase the proportion of people without vomiting (RR 1.11, 95% CI 0.86 to 1.44; *I* ^2^ = 0%; 4 EC, 209 participants)	Moderate	High
		Absence of nausea and vomiting: cannabinoids may increase the proportion of people without nausea and vomiting (RR 2.0; 95% CI 0.74 to 5.4; *I* ^2^ = 60%; EC, 414 participants)	Low	High
		Study dropout due to adverse events: cannabinoids may increase the proportion of people who drop out of the study for this reason (RR 3.9; 95% CI 1.25 to 12.2; *p* = 0.02; *I* ^2^ = 17%; 5 EC, 664 participants)	Low	High
Sleep disorders ‐ Any disorder	Dronabinol (different doses, regimens, and durations) versus placebo	Sleep pattern (different tools): SMD 0.73; 95% CI −0.39 to 0.12; 7 EC, 683 participants	Low	Critically low
Ulcerative colitis	CBD + THC (up to 4.7%) 100 mg to 500 mg/day, oral capsules versus placebo, 10 weeks	Clinical remission (Mayo score < 2): CBD + THC may reduce the risk of remission (RR 0.94; 95% CI 0.39 to 2.25; 1 EC; 60 participants)	Low	High
		Clinical response (reduction in Mayo score ≥ 3): CBD + THC may increase the likelihood of response (RR 1.37, 95% CI 0.59 to 3.21; 1 EC; 60 participants)	Low	High
		Quality of life (IBDQ): CBD + THC likely improves quality of life (SMD 17.4, 95% CI −3.45 to 38.25 1 EC, 53 participants)	Moderate	High
		Serious adverse events: CBD + THC may reduce the risk of serious adverse events (RR 0.15, 95% CI 1.05 to 1.56; 1 EC, 60 participants)	Low	High
		Adverse events: CBD + THC probably increases the risk of adverse events (RR 1.28, 95% CI 0.01 to 2.83; 1 EC, 60 participants)	Moderate	High
	Cigarette containing 0.5 mg of cannabis, twice daily (up to 23 mg THC/day) versus placebo, 8 weeks	C‐reactive protein (CRP, serum levels, mg/L): cannabis smoking may slightly reduce CRP levels (MD: −0.3; 95% CI −1.35 to 0.75; 1 RCT, 28 participants)	Low	High

## 5 Discussion

This overview included 102 systematic reviews that assessed the effects of cannabis derivatives and their synthetic analogues for therapeutic purposes for any health condition. The summarised results with available certainty of evidence assessment are presented in Table 1, throughout the main document, while extracted results for all clinical conditions details are in Supporting Information S5.

Considering the results of the outcomes for which there is an assessment of the certainty of the evidence, this overview points to benefits of cannabis sp. for the following conditions:

- Chronic non‐cancer pain—different dosages and presentations of cannabinoids probably reduce pain scores, although without reaching the minimum clinically relevant difference (moderate certainty of evidence).
- Crohn's disease—cannabis sp. oil may improve quality of life (low certainty of evidence).
- Multiple sclerosis—different dosages and presentations of cannabinoids are likely to increase the frequency of participants with significant reduction in spasticity and reporting “great” or “very great” improvement (but may have little or no effect on quality of life and may increase the risk of total adverse events, nervous system adverse events, psychiatric disorders and early treatment discontinuation).
- Post‐chemotherapy nausea and vomiting—different dosages and presentations of cannabinoids are likely to increase the proportion of people without nausea and vomiting.
- Ulcerative colitis—the combination of CBD + THC likely improves quality of life, may increase the likelihood of remission and clinical response, and may reduce the risk of serious adverse events (but likely increases the risk of any adverse events).

Considering the results of the outcomes for which there is an assessment of the certainty of the evidence, this overview points to the absence of benefits of cannabis sp. for the following conditions:

- Cancer pain in people receiving palliative care—CBD oil may not increase the proportion of people with significant pain improvement.
- Cancer pain related to radiotherapy—nabilone may not increase the proportion of people with significant pain improvement (low certainty of evidence).
- Neuropathic cancer pain—nabilone may not increase the proportion of people with significant pain improvement and may increase the risk of central nervous system adverse events.
- Opioid‐refractory cancer pain—nabiximols probably does not increase the overall perception of improvement, may not reduce the daily dose of opioids (but probably does not increase the risk of serious adverse events or treatment discontinuation).
- Sleep disturbances—dronabinol may not improve sleep patterns.

The quality of systematic reviews, assessed using the AMSTAR‐2 tool, indicates the robustness of the methods used to synthesise the results of each systematic review and should therefore be considered in the decision‐making process. From the 102 included systematic reviews, 68.6% were considered to be of critically low quality and only 17.6% of high quality. Thus, most systematic reviews were of low or critically low quality. This finding was reflected in the presentation of the results, preventing detailed data on the population, cannabinoid, and outcomes from being presented clearly, reducing external validity and applicability. In fact, reviewers from this overview often had to access the primary studies to obtain or confirm results that were insufficiently presented in the included reviews.

Although the highest quality systematic reviews identified in this overview were conducted by Cochrane, all are outdated. Given the publication of recent studies, the findings of these reviews may no longer reflect the totality of available evidence, which may influence clinical interpretations and public health policy decisions.

The assessment of certainty of evidence using the GRADE approach is an important aspect of supporting decision‐making, as it allows for estimating how reliable the numerical results are, facilitates the interpretation of results, and enables the development of conclusions and recommendations in a standardised manner. The certainty of evidence on efficacy was assessed for only 59% of systematic reviews and was not assessed by any systematic review that focused exclusively on safety.

For one of the reviews included on the effects of cannabinoids on Alzheimer's disease (Ruthirakuhan et al. 2019), the full text could not be retrieved despite repeated attempts. The systematic review was included as it fulfilled the inclusion criteria, but methodological quality assessment and outcome extraction were not performed. The impact on the synthesis was limited because a more recent systematic review of high methodological quality according to AMSTAR‐2 was available for the same condition (Kuharic et al. 2021) and was used to extract outcome‐level data (Kuharic et al. 2021).

Another phenomenon observed when analysing the systematic reviews included in this overview was the overlap of diseases and symptoms, i.e., the overlap in how studies group participants. In some cases, the criteria are based on specific diseases (clinical diagnoses) and, in others, on isolated symptoms, as occurred in conditions as chronic pain and depressive disorders, for example. This approach tends to compromise the comparability of results, increase heterogeneity, and reduce the clinical applicability of evidence, in addition to potentially increase the risk of distortion in the interpretation of data (spin bias).

Another important point concerns the rule applied to compose the outcome synthesis when multiple systematic reviews addressed the same condition. Selecting the review with the highest AMSTAR‐2 rating and the most recent search date prioritises the review most likely to be used in clinical decision‐making but does not guarantee that all RCTs available across redundant reviews are captured in the synthesis. For conditions with several available reviews, particularly chronic pain, multiple sclerosis, and post‐chemotherapy nausea and vomiting, alternative review choices could, in principle, modify the body of RCTs underlying the synthesis. Supporting Information S5 lists, for each condition, all reviews considered, allowing readers to consult the alternative sources directly.

Safety outcomes were not reported in a uniform way by the included systematic reviews. Most reviews did not provide detailed information on specific adverse events, and no review that focused exclusively on safety used the GRADE approach to assess certainty of evidence. This limits the ability of an overview to characterise the safety profile of cannabis derivatives at the level of detail required for clinical decision‐making.

This overview was conducted following the methodological guidelines of the Cochrane Handbook for Systematic Reviews of Interventions (Higgins et al. 2024) and the relevant items of PRISMA 2020 (Page et al. 2021) were adopted for the preparation of the final article. A broad and sensitive search was used to identify eligible SRs. Detailed data extraction and access to primary studies were performed to increase the accuracy of the information. This helped to ensure the applicability of the results in practice and to guide future studies.

An overview of systematic reviews from 2022, prepared by the authors of this overview, identified 67 systematic reviews, pointing out that over 3 years, 35 new eligible reviews were published, showing an increase of more than 50% in the volume of systematic reviews on the effects of cannabis derivatives and their synthetic analogues during this period. This highlights the need for periodic updates to this overview to ensure that recommendations remain based on robust and recent data.

As implications for future research, this overview opens up a broad and complex field for the development of primary studies to evaluate the effects of cannabinoids as primary or adjunctive therapy for different health conditions and reinforces the importance of safety assessment.

Furthermore, these results reinforce the need for initiatives to improve the planning, conduct, and reporting of systematic reviews in this area so that they can provide more reliably support the development of public policies on the production, development, construction of scientific knowledge, and use of cannabinoids in healthcare.

As an implication for practice, decision‐makers and guideline developers can be guided by the results summarised in Table 2 and Supporting Information S5.

However, when making formal recommendations, it is essential to consider: (i) the quality of the systematic review that mapped the evidence (classification of the systematic review by AMSTAR‐2), and (ii) the certainty of the evidence identified for each outcome.

## 6 Conclusion

This overview included 102 systematic reviews, 68.6% of which were of critically low quality and 17.6% were of high quality, that evaluated the effects of cannabis derivatives and their synthetic analogues for therapeutic purposes. There is low to moderate certainty of evidence of benefits from these interventions for ulcerative colitis, chronic non‐cancer pain, Crohn's disease, multiple sclerosis, and post‐chemotherapy nausea and vomiting; however, the results point to a lack of benefits for sleep disorders, chronic cancer pain refractory to opioids, pain related to radiotherapy, and pain in people receiving palliative care.

For other health conditions and outcomes or comparisons, the certainty of the evidence was very low or not assessed, which limits concrete recommendations regarding the routine use of these compounds. Further studies are needed to reduce uncertainties about the therapeutic use of cannabis sp. derivatives and their synthetic analogues.

## Author Contributions

**Marco Aurelio de Carvalho Nascimento:** conceptualization, funding acquisition, writing – original draft, project administration. **Daniela Fortunato Rêgo:** conceptualization, funding acquisition, writing – original draft, formal analysis, project administration. **Rachel Riera:** conceptualization, methodology, investigation, funding acquisition, writing – review and editing, project administration, data curation, supervision, validation. **Carolina de Oliveira Cruz Latorraca:** conceptualization, writing – original draft, methodology, formal analysis. **Rafael Leite Pacheco:** conceptualization, investigation, writing – review and editing, writing – original draft, funding acquisition, methodology, formal analysis, software, visualization. **André Bastos Daher:** conceptualization, funding acquisition, writing – original draft, project administration.

## Funding

This work was supported by Fundação Oswaldo Cruz.

## Conflicts of Interest

The authors declare no conflicts of interest.

## Supporting information


**Data S1:** Search strategies for each database and search results obtained on 9 April 2025.

**Data S2:** Studies excluded (after reading the full text) and reasons for exclusion, considering the eligibility criteria of this overview.

**Data S3:** Main characteristics of the included systematic reviews.

**Data S4:** Complete assessment of the included systematic reviews using the AMSTAR‐2 tool.

**Data S5:** Results of the efficacy and safety outcomes assessed for each health condition, considering the systematic reviews with the best methodological quality and/or most recent searches.

## Data Availability

The data that supports the findings of this study are available in the Supporting Information of this article.
