# Supplementary material for: Efficacy and Safety of Cannabis Derivates and Their Synthetic Analogs. Overview of Systematic Reviews
Source: Phytother Res. 2026 Jun 23;40(11):6112–29. doi: 10.1002/ptr.70403 (PMC13753713; doi:10.1002/ptr.70403)
Supplement: Supplementary file 1 [file PTR-40-6112-s001.docx]

Supplementary material 5

Results of the efficacy and safety outcomes assessed for each health condition, considering the systematic reviews with the best methodological quality and/or most recent searches.

Acute pain *(Barakji 2023 – low quality; Cardenas 2025 – critically low quality; Votrubec 20221 – high quality)*

Any acute pain

*Cannabinoids (different dosages and presentations) versus placebo*

- Pain (ENA): the effects of nabilone are uncertain (MD 0.52; 95% CI -0.40 to 1.43; p = 0.19; I² = 90%; 5 RCTs, 530 participants).

Low back pain

*CBD 400 mg versus placebo, single dose*

- Pain after 2 hours (ENA, score from 0 to 10): MD - 0.3; 95% CI –1.3 to 0.6; 1 RCT, 100 participants.

Amyotrophic lateral sclerosis *(Bilbao 2022, Belgers 2023 - critically low quality)*

*Dronabinol 10 mg once daily versus placebo, 2 weeks*

- Quality of life (ALSAQ-40): MD -0.09; 95% CI -0.41 to 0.23; 1 cross-over RCT, 27 participants.
- Anxiety/depression (HADS): MD -0.15; 95% CI -0.66 to 0.36; 1 cross-over RCT, 27 participants.

*CBD 30 mg + THC 32.4 mg once daily (nabiximols) versus placebo, 6 weeks*

- Spasticity (change in Modified Ashworth Scale): MD -0.32; 95% CI -0.57 to -0.069; p = 0.01; 1 RCT, 59 participants.
- Pain (change in ENA): MD -1.15; 95% CI -2.10 to -0.21; p = 0.017; 1 RCT, 59 participants.
- Spasm frequency (change in ENA): MD 0.71; 95% CI -1.68 to 0.27; p = 0.153; 1 RCT, 59participants.
- Activities of daily living (change in Activities of Daily Living Index): MD 0.73; 95% CI -2.56 to 4.02; p = 0.666; 1 RCT, 59 participants.

Anxiety disorders *(Whiting 20151 – moderate quality; Coelho 2024; Kock 2024 – critically low quality)*

Social anxiety disorder

*CBD 300 mg versus placebo, 6 months*

- Avoidance level (Fear Questionnaire - FQ, score 0 to 120): no difference between groups (MD 10.11; 95% CI -0.81 to 21.03; p = 0.07; 1 EC, 37 participants).
- Anxiety severity (Beck Inventory Scale - BAI, score 0 to 63): no difference between groups (MD 3.86; 95% CI -1.76 to 9.48; p = 0.18; 1 RCT, 37 participants).

Attention deficit hyperactivity disorder *(Bilbao 2022 - critically low quality)*

*Nabiximols (Sativex) versus placebo, 4 weeks*

- Hyperactivity and impulsivity: SMD -0.83, 95% CI -1.58 to -0.09; p = 0.03; 1 RCT, 30 participants.

Autism spectrum disorder *(Kock 2024 - critically low quality)*

*CBD 420 mg + THC 21 mg (whole plant) versus placebo, 12 weeks*

- Behaviour in daily activities (HSQ-ASD): DMP 0.41; 95% CI -0.037 to 0.855; 1 RCT, 79 participants.

*CBD 420 mg + THC 21 mg (extract) versus placebo, 12 weeks*

- Behaviour in daily activities (HSQ-ASD): SMD 0.12, 95% CI -0.316 to 0.56; 1 RCT, 81 participants.

*CBD 17.5 mg + THC 1.95 mg versus placebo, 12 weeks*

- Treatment response (ATEC-T): SMD 0.335; 95% CI -0.176 to 0.845; 1 RCT, 60 participants.
- Autism Symptom Scale (CARS): SMD 0.492; 95% CI -0.022 to 1.006; 1 RCT, 60 participants.

Bipolar disorder *(Bonaccorso 2019 - critically low quality)*

*CBD 300 mg/day versus placebo, 8 weeks**

- Psychosocial function (FAST): MD 0.52; 95% CI -10.1 to 11.2; p = 0.92; 1 RCT, 35 participants.
- Clinical Global Impression (CGI-S): MD 0.52; 95% CI -0.59 to 1.63; p = 0.35; 1 RCT, 35 participants.
- Global clinical impression (BPRS): MD 0.52; 95% CI -0.59 to 1.63; p = 0.35; 1 RCT, 35 participants.
- Quality of life (PHQ-9): MD -0.67; 95% CI -5.49 to 4.16; p = 0.78; 1 RCT, 35 participants.
- Anxiety symptoms (HAMA): MD -0.51; 95% CI -5.55 to 4.54; p = 0.84; 1 RCT, 35 participants.
- Depressive symptoms (change in MADRS score between baseline and 8 weeks): MD -0.82; 95% CI -7.81 to 6.17; p = 0.81; 1 RCT, 35 participants.
- Depressive symptoms (HAMD): MD -2.47; 95% CI -7.24 to 2.30; p = 0.31; 1 RCT, 35 participants.

**Study terminated prematurely due to the coronavirus pandemic before the full sample could be recruited.*

Cachexia syndrome, anorexia, cancer-related loss of appetite *(Alderman 2023 – critically low quality; Wang 20191 – moderate quality)*

*THC 2.5 mg or THC 2.7 mg + CBD 1 mg versus placebo, 19 to 42 weeks*

- Appetite: MD 0.27; 95% CI -0.51 to 1.04; 3 RCTs, 592 participants.
- Quality of life: MD -12.39; 95% CI -24.21 to -0.57; 2 RCTs, 223 participants.

**starting with 0.5 mg for 2 weeks followed by 1 mg for 6 weeks.*

Cannabis dependence *(Vuilleumier 20221 – critically low quality)*

*Nabilone capsules 2 mg/day versus placebo, 10 weeks**

- Adherence to treatment (considering the total number of possible doses): 97% with nabilone versus 95.6% with placebo (RR 1.01; 95% CI and p-value not reported; 1 RCT, 18 participants).

**starting with 0.5 mg and increasing by 0.5 mg each week to a dose of 2 mg/day for 4 weeks, followed by a gradual reduction over the last 3 weeks, reversing the titration schedule.*

*Nabiximols (Sativex® spray, THC 2.7 mg + CBD 2.5 mg) up to 32 doses/day versus placebo, 1 week**

- Treatment adherence (time to non-adherence): greater with nabiximols (HR 3.66; 95% CI 1.18 to 11.37; p = 0.02; 1 RCT, 86 participants).

*32 doses/day: THC 86.4 mg + CBD 80 mg*

*Nabiximols (Sativex® spray, THC 2.7 mg + CBD 2.5 mg) average of 9.5 doses/day versus placebo, 12 weeks**

- Cannabis consumption (days): lower in the nabiximols group (MD 18 days; 95% CI 3.5 to 33.7; p = 0.02; 1 RCT, 128 participants).

*17.6 doses/day: THC 47.5 mg + CBD 44 mg*

*CBD 400 mg or 800 mg/day versus placebo, 4 weeks **

- Abstinence (number of days without cannabis use): 400 mg - MD 0.48 days; 95% CI 0.15 to 0.82; 1 EC, 24 participants; certainty of evidence not assessed; 800 mg - MD 0.27 days; 95% CI -0.09 to 0.64; 1 RCT, 24 participants.
- Withdrawal symptoms (Cannabis Withdrawal Scale): after 4 weeks of treatment, symptoms were lower with CBD 800 mg (MD -1.26; 95% CI -2.13 to -0.39; 1 RCT, 24 participants, but not with CBD 400 mg (MD -0.34; 95% CI -1.14 to 0.50; 1 RCT, 24 participants). After the 24-week follow-up period, symptoms were lower with CBD 800 mg (MD -2.5; 95% CI -3.08 to -1.93; 1 RCT, 24 participants), and with CBD 400 mg (MD -1.32; 95% CI -1.89 to -0.06; 1 RCT, 24 participants).

**The CBD 200 mg arm was discontinued due to ineffectiveness during the study.*

Chronic pain *(Fischer 2021; Hauser 2023; Wang 20211 – high quality)*

Opioid-refractory cancer pain

*Nabiximols (Sativex® spray, THC 2.7 mg + CBD 2.5 mg, average of 6.3 to 11 doses/day) versus placebo*

- Pain (improvement or much improvement in PGIC): cannabinoids probably do not improve PGIC (RR 0.06; 95% CI 0.01 to 0.12; 4 RCTs, 996 participants).
- Pain (≥ 30% improvement): RR 0.02; 95% CI -0.03 to 0.07; 4 RCTs, 1332 participants.
- Pain (improvement ≥ 50%): RR 0.01; 95% CI -0.02 to 0.05; 4 RCTs, 1333 participants.
- Maintained average daily opioid dose: cannabinoids may not alter the daily dose of opioids (SMD 0.08; 95% CI -0.10 to 0.27; 3 RCTs, 970 participants).
- Sleep disturbance: SMD -0.06; 95% CI -0.19 to 0.06; 4 RCTs, 1314 participants.
- Serious adverse events: cannabinoids probably do not alter the risk of serious adverse events (RR 0.02; 95% CI -0.03 to 0.07; 4 EC, 1330 participants).
- Dropout due to adverse events: cannabinoids probably do not alter the risk of dropout (RR 0.04, 95% CI 0.0 to 0.08; 4 EC, 1332 participants).

Any chronic non-cancer pain

*Cannabinoids (different dosages and presentations) versus placebo, 1 to 4 weeks*

- Pain (VAS): cannabinoids probably reduce VAS scores, but this reduction does not reach the minimum clinically relevant difference (SMD -0.63, 95% CI -0.96 to -0.29; 23 RCTs, 2,580 participants).
- Pain (≥ 30% reduction, VAS): RR 1.23; 95% CI 0.98 to 1.53; 9 EC.

Crohn's disease *(Kang 2025 – critically low quality; Kafil 2018b – high quality)*

*Cannabinoids (different dosages and presentations) versus placebo*

- Disease activity index (CDAI): SMD -0.91; 95% CI -1.54 to -0.28; p = 0.007; I^2^ = 71.9%; 5 RCTs, 172 participants.
- Endoscopic activity index (SES-CD): SMD -0.29; 95% CI -0.68 to 0.10; p = 0.43; I^2^ = 0%; 2 RCTs, 102 participants.
- Quality of life (SF-36): SMD 1.86; 95% CI 0.55 to 3.17; I^2^ = 0%; 3 RCTs, 132 participants.

*Cannabis sp. cigarette (115 mg THC) twice daily versus cannabis cigarette without THC, 8 weeks*

- Clinical remission (CDAI score < 150): the effects of cannabis cigarette with THC are very uncertain (RR 4.55, 95% CI 0.63 to 32.56; 1 EC, 21 participants).
- Clinical response (CDAI reduction ≥ 100): the effects of cannabis cigarettes with THC are very uncertain (RR 2.27, 95% CI 1.04 to 4.97; 1 RCT, 21 participants).
- Adverse events: the effects of THC cannabis cigarettes are very uncertain (RR 4.09; 95% CI 1.15 to 14.57; 1 RCT, 21 participants).

*Cannabis sp. oil (CBD 5%, sublingual use) 2 ml twice daily versus placebo for 8 weeks*

- Clinical remission (CDAI score ≤ 150): the effects of cannabis sp. oil are very uncertain (RR 1.20, 95% CI 0.36 to 3.97; 1 EC, 19 participants).
- Serious adverse events: the effects of cannabis sp. oil are very uncertain (RR 0.90, 95% CI 0.07 to 12.38; 1 RCT, 19 participants).

*Cannabis oil (CBD 15% and THC 4%, sublingual use) versus placebo, 8 weeks*

- Quality of life (SF-36): cannabis oil sp. may increase SF-36 scores (MD 16.4, 95% CI 5.72 to 27.08; 1 RCT, 39 participants.

Dementia *(Kuharic 2021^60^ - high quality)*

*Nabilone 0.5 mg/day to 2 mg/day versus placebo, 6 weeks*

- Cognitive function (Mini-Mental): no clinically relevant difference between groups, but this result is very uncertain (MD 1.10, 95% CI 0.10 to 2.10; 1 cross-over RCT, 38 participants).
- Psychological/behavioural symptoms (NPI-NH scale): may be better with nabilone (MD -4.6; 95% CI -7.5 to -1.7; 1 crossover RCT, 38 participants).

*Natural THC (delta THC 98%) or dronabinol or nabilone versus placebo, 3 to 14 weeks*

- Psychological/behavioural symptoms (NPI-NH or NPI, score from 0 to 144; the higher, the more symptoms): MD -1.97; 95% CI -3.87 to 0.07; 3 RCTs, 111 participants.
- Agitated or aggressive behaviour (CMAI, score from 29 to 203; higher score indicates more symptoms): MD -2.35; 95% CI -4.10 to -0.60; 3 RCTs, 111 participants.
- General adverse events: OR 0.70; 95% CI 0.11 to 4.38; 1 RCT, 50 participants.

Epilepsy – Dravet syndrome *(Lattanzi 2020 – low quality; Oliveira 2025 – critically low)*

*CBD 10mg/kg/day versus placebo, 14 weeks*

- Reduction in seizure frequency (participants with ≥ 50% reduction in monthly seizure frequency from baseline): RR 1.68; 95% CI 1.03 to 2.75; 1 EC, 131 participants.
- Total adverse events: RR 0.97; 95% CI 0.86 to 1.10; p = 0.617; 1 RCT, 131 participants.
- Serious adverse events: RR 1.36; 95% CI 0.67 to 2.76; p = 0.394; 1 RCT, 131 participants.

*CBD 20mg/kg/day versus placebo, 4 weeks*

- Reduction in seizure frequency (participants with ≥ 50% reduction in monthly seizure frequency from baseline): RR 1.73; 95% CI 1.22 to 2.45; 2 RCTs, 252 participants.
- Total adverse events: RR 1.08; 95% CI 0.98 to 1.18; p = 0.122; 2 RCTs, 252 participants.
- Serious adverse events: RR 1.81; 95% CI 1.00 to 3.29; p = 0.051; 2 RCTs, 252 participants.

Epilepsy – Lennox-Gastaut syndrome *(Lattanzi 2018a– low quality; Oliveira 2025 – critically low)*

*CBD 10mg/kg/day versus placebo, 14 weeks*

- Reduction in seizure frequency (participants with ≥ 50% reduction in monthly seizure frequency from baseline): RR 1.73; 95% CI 1.22 to 2.45; 2 RCTs, 252 participants.
- Adverse events: RR 1.16; 95% CI 0.97 to 1.38; p = 0.106; 1 RCT, 143 participants.

*CBD 20mg/kg/day versus placebo, 14 weeks*

- Reduction in seizure frequency (participants with ≥ 50% reduction in seizure frequency from baseline): RR 1.62; 95% CI 1.09 to 2.43; 1 RCT, 156 participants.
- Adverse events: RR 1.27; 95% CI 1.14 to 1.342; p < 0.001; 2 RCTs, 329 participants.

Fragile X syndrome *(Chhabra 2024 - critically low quality)*

*CBD (transdermal gel) 250 mg/day if weight ≤ 35 kg and 500 mg/day if weight >35 kg versus placebo, 12 weeks*

- Overall adverse events: 50% with placebo versus 57.8% with CBD (RR 1.16, 95% CI 0.90 to 1.49; 1 RCT, 212 participants).

HIV/AIDS *(Lutge 2013 - high quality)*

*Dronabinol 5 mg/day versus placebo, 6 weeks*

- Body weight (frequency of participants with weight gain ≥ 2 kg): RR 2.4; 95% CI 0.7 to 8.23; 1 RCT, 88 participants.
- Nausea and vomiting (frequency of participants with improvement): 16/72 dronabinol versus 3/67 placebo (RR 4.96; 95% CI 1.51 to 16.27; p = 0.01; 1 RCT, 88 participants).
- Mood (frequency of participants with improvement): 5/72 with dronabinol versus 1/67 with placebo (OR 4.93; 95% CI 0.56 to 43.3; p = 0.15; 1 RCT, 139 participants).
- Adverse events (frequency of participants with ≥ one adverse event): 4% with dronabinol versus 13% with placebo (RR 3.3; 95% CI 1.65 to 6.22; p = 0.0006; 1 RCT, 139 participants).

Huntington's disease *(Belgers 2023; Oikonomou 2022 - critically low quality)*

*Nabilone 1 mg or 2 mg versus placebo, 5 weeks*

- Motor ability (UHDRS): MD 0.86; 95% CI -1.8 to 3.52; p = 0.5; 1 cross-over RCT, 22 participants.
- Chorea severity (UHDRS): MD 1.68; 95% CI 0.44 to 2.92; p = 0.009; 1 cross-over RCT, 22 participants.
- Cognition (UHDRS): MD 3.57; 95% CI -3.41 to 10.55; p = 0.30; 1 cross-over RCT, 22 participants.
- Behaviour: MD 4.01; 95% CI -0.11 to 8.13; p = 0.06; 1 cross-over RCT, 22 participants.
- Neuropsychiatric inventory: MD 6.43; 95% CI 0.2 to 12.66; p = 0.04; 1 cross-over RCT, 22 participants.

*Nabiximols (Sativex® spray, THC 2.7 mg + CBD 2.5 mg) up to 12 doses/day versus placebo, 12 weeks**

- Quality of life (HADS-D): MD -0.11; 95% CI -0.43 to 0.2; 1 cross-over RCT, 26 participants.

*12 doses/day: THC 32.4 mg + CBD 30 mg*

Irritable bowel syndrome *(Bilbao 2020 - critically low quality)*

*Dronabinol 2.5 mg versus dronabinol 5 mg versus placebo, single dose*

- Symptoms of the disease: the effects are uncertain (SMD -0.53; 95% CI -1.06 to 0.00; 1 RCT, 75 participants).

Knee osteoarthritis *(Wong 2020 – critically low quality)*

*Endocannabinoid PF-04457845 (FAAH inhibitor) versus placebo, 2 weeks**

- Pain (WOMAC): MD 0.04; 80% CI -0.63 to 0.71; p = 0.94; 1 RCT, 107 participants.

**Study terminated early in the interim analysis due to futility.*

Multiple sclerosis *(Filippini 2022 - high quality)*

*Cannabinoids (different dosages and presentations) versus placebo, 4 to 48 weeks*

- Spasticity (frequency of participants reporting ≥ 30% reduction in spasticity, NNT): cannabinoids probably increase the frequency of participants with reduced spasticity (odds ratio [OR] 2.51; 95% CI 1.56 to 4.04; p = 0.0002; 5 RCTs, 1,143 participants).
- Pain (frequency of participants reporting ≥ 50% reduction in pain, NNT): effects are uncertain (OR 4.23; 95% CI 1.11 to 16.17; 1 RCT, 48 participants.
- Quality of life: cannabinoids may have little or no effect on quality of life (SMD -0.08; 95% CI -0.17 to 0.02; 8 RCTs, 1942 participants; I² = 0%).
- Perceived overall improvement: cannabinoids probably increase the number of people reporting a “very large” or “large” improvement in health status compared with placebo (OR 1.80; 95% CI 1.37 to 2.36; 8 EC, 1215 participants; I² = 0%).
- Total adverse events: may be more frequent with cannabinoids (OR 1.38; 95% CI 0.96 to 1.99; 20 EC, 3,124 participants; I^2^ = 0%).
- Adverse events of the nervous system: may be more frequent with cannabinoids (OR 2.61; 95% CI 1.53 to 4.44; 7 EC, 1,154 participants; I^2^ = 63%).
- Psychiatric disorders: may be more frequent with cannabinoids (OR 1.94; 95% CI 1.31 to 2.88; 6 EC, 1,122 participants; I^2^ = 0%;).
- Treatment discontinuation due to adverse events: may be more frequent with cannabinoids (OR 2.41; 95% CI 1.51 to 3.84; 21 EC, 3,110 participants; I^2^ = 17%).

Parkinson's disease *(Belgers 2023; Bilbao 2022 - critically low quality)*

*Nabilone 2 mg/day versus placebo, 4 weeks*

- Overall motor/non-motor assessment (MDS-UPDRS-I): the effects of nabilone are uncertain (MD 1.63; 95% CI 0.09 to 3.18; p = 0.03; 1 RCT, 38 participants; very low certainty of evidence).
- Anxiety (HADS-A): the effects of nabilone are uncertain (MD -0.08; 95% CI -0.72 to 0.56; 1 RCT, 38 participants).
- Pain (VAS): the effects of nabilone are uncertain (MD -4.42; 95% CI -0.02 to 11.17; p = 0.96; 1 RCT, 38 participants).
- Cognition (MoCA): the effects of nabilone are uncertain (MD -0.74; 95% CI -1.81 to 0.46; p = 0.053; 1 RCT, 38 participants).
- Fatigue (FSS): the effects of nabilone are uncertain (MD 4.00; 95% CI -3.53 to 11.453; p = 0.36; 1 RCT, 38 participants).
- Sleep pattern (ESS): the effects of nabilone are uncertain (MD -0.05; 95% CI -1.5 to 1.45; p = 0.91; 1 RCT, 38 participants).
- Quality of life (PDQ-39): the effects of nabilone are uncertain (MD 0.03; 95% CI -3.59 to 3.64; p = 0.907; 1 RCT, 38 participants).

Post-chemotherapy nausea and vomiting *(Chow 2025 critically low quality; Smith 2015 – high quality)*

Cannabinoids versus placebo

*Cannabinoids (different dosages and presentations) versus placebo*

- Absence of nausea and vomiting: cannabinoids probably increase the proportion of people without nausea and vomiting (RR 2.9; 95% CI 1.8 to 4.7; 3 EC, 288 participants).
- Absence of vomiting: cannabinoids may increase the proportion of people without vomiting (RR 5.7; 95% CI 2.6 to 12.6; 3 RCTs, 168 participants).
- Study withdrawal due to adverse events: the effects of cannabinoids on the risk of withdrawal are uncertain (RR 6.9; 95% CI 1.96 to 24; 2 RCTs, 276 participants).
- Total control of nausea and vomiting: greater with cannabinoids (RR 2.65; 95% CI 1.70 to 4.12; 8 RCTs, 406 participants).

*Dronabinol 50 mg/day/m² versus placebo, 1 day (divided into 5 doses)*

- Absence of nausea: no difference between groups (RR 2.0; 95% CI 0.2 to 21; 2 RCTs, 96 participants).
- Absence of vomiting: no difference between groups (RR 3.0; 95% CI 0.65 to 13.76; 2 RCTs, 96 participants).

*Nabilone 2 mg/day versus placebo during two cycles of chemotherapy*

- Absence of vomiting: higher proportion of people without vomiting with nabilone (RR 7.25, 95% CI 2.84 to 18.52; 1 RCT, 72 participants).

Cannabinoids versus antiemetics

*Cannabinoids (different dosages and presentations) versus different antiemetics*

- Dizziness: greater with cannabinoids (RR 2.54; 95% CI 1.91 to 3.37; I² = 18%; 9 EC, 743 participants).

*Cannabinoids (different dosages and presentations) versus prochlorperazine (different dosages and presentations)*

- Absence of nausea: cannabinoids may increase the proportion of people without nausea (RR 1.46; 95% CI 0.67 to 3.15; I² = 58%; 5 EC, 258 participants).
- Absence of vomiting: cannabinoids probably increase the proportion of people without vomiting (RR 1.11; 95% CI 0.86 to 1.44; I^2^ = 0%; 4 EC, 209 participants).
- Absence of nausea and vomiting: cannabinoids may increase the proportion of people without nausea and vomiting (RR 2.0; 95% CI 0.74 to 5.4; I^2^ = 60%; RCT, 414 participants).
- Study withdrawal due to adverse events: cannabinoids may increase the proportion of people who withdraw from the study due to adverse events (RR 3.9; 95% CI 1.25 to 12.2, p = 0.02, I^2^ = 17%; 5 RCTs, 664 participants).
- Total control of nausea and vomiting: no difference between groups (RR 1.52; 95% CI 0.79 to 2.90; 11 RCTs, 550 participants.
- Dizziness: greater with cannabinoids (RR 2.36; 95% CI 1.82 to 3.07; I² = 12%; 7 EC, 675 participants).

*Dronabinol (different dosages and presentations) versus prochlorperazine (different dosages and presentations)*

- Absence of nausea: no difference between groups (RR 2.38; 95% CI 0.21 to 26.91; I² = 68%; 2 RCTs, 117 participants).
- Absence of vomiting: no difference between groups (RR 1.05; 95% CI 0.64 to 1.71; I² = 0%; 2 RCTs, 116 participants).
- Absence of nausea and vomiting: no difference between groups (RR 1.44; 95% CI 0.62 to 3.31; I² = 52%; 3 RCTs, 188 participants).

*Levonantradrol 0.5 mg to 1 mg/4 doses versus chlorpromazine 25 mg/4 doses, 1 day*

- Total control of nausea and vomiting: no difference between groups (RR 1.19; 95% CI 0.63 to 2.23; 1 EC, 108 participants).

*Nabilone (different dosages and presentations) versus prochlorperazine (different dosages and presentations)*

- Absence of nausea: no difference between groups (RR 1.41; 95% CI 0.33 to 6.03; I² = 53%; 3 RCTs, 141 participants).
- Absence of vomiting: no difference between groups (RR 1.55; 95% CI 0.39 to 126.48; I² = 35%; 2 RCTs, 93 participants).
- Absence of nausea and vomiting: greater with nabilone (RR 17; 95% CI 0.99 to 291.06; 1 RCT, 226 participants).
- Hallucinations: no difference between groups (RR 5.39; 95% CI 0.66 to 43.68; I² = 0%; 2 RCTs, 144 participants).

*Nabilone 1 mg three times daily versus domperidone 20 mg three times daily during two cycles of chemotherapy*

- Study dropout due to adverse events: no difference between groups (RR 3.00; 95% CI 0.13 to 71.40; 1 RCT, 76 participants).
- Dizziness: greater with nabilone (RR 2.75; 95% CI 1.06 to 7.12; 1 RCT, 8 participants).

*Nabilone 2 mg/day versus alizapride 150 mg/day, 5 days*

- Total control of nausea and vomiting: no difference between groups (RR 1.99; 95% CI 0.58 to 6.82; 1 cross-over RCT, 20 participants).

*THC 10 mg/8 doses versus haloperidol 2 mg/8 doses, 1 day*

- Total control of nausea and vomiting: no difference between groups (RR 1.09, 95% CI 0.65 to 1.84; 1 RCT, 52 participants).

*THC 2.5 mg to 5 mg/day versus metoclopramide 5 to 10 mg/day, 1 day (different dosages and presentations)*

- Total control of nausea and vomiting: no difference between groups (RR 1.80; 95% CI 0.81 to 4.0; 1 RCT, 19 participants).

Rheumatoid arthritis *(Wong 2020^116^ - low quality)*

*Nabiximols (Sativex® spray, THC 2.7 mg + CBD 2.5 mg) up to 5 doses/day versus placebo, 5 weeks*

- Pain on movement (numerical pain scale): MD 0.95; 95% CI -1.83 to 0.02; p = 0.04; 1 RCT, 58 participants.

*5 doses/day: THC 13.5 mg + CBD 12.5 mg*

Schizophrenia and other psychotic disorders *(Kock 2024 critically low; McLoughlin 2014 – high quality)*

*CBD 800 mg versus amisulpride 800 mg, 4 weeks**

- Mental state (BPRS, 0–126; higher score indicates poorer mental health): MD 1.10; 95% CI –8.18 to 10.38; 1 RCT, 35 participants.
- Disease severity (PANSS): MD 0.40; 95% CI -13.42 to 14.22; 1 RCT, 35 participants.

*Starting with 200 mg and gradually increasing to 800 mg in the first week for both interventions.*

Sleep disorders *(Aminilari 2021 – low quality; Bilbao 2022 – critically low quality; Whiting 2015 – moderate quality)*

Any sleep disturbance

*Dronabinol (different doses, regimens, and duration) versus placebo*

- Sleep pattern (different tools): SMD 0.73, 95% CI -0.39 to 0.12; 7 RCTs, 683 participants.

*Dronabinol 7.5 mg to 30 mg/day versus diazepam 3 to 15 mg/day*

- Sleep pattern (different tools): SMD 1.12, 95% CI 0.37 to 1.87; 1 cross-over RCT, 22 participants.

*Nabilone (different doses, regimens, and durations) versus placebo*

- Sleep pattern (different tools): SMD -0.58, 95% CI -1.14 to -0.01; I² = 77%; 6 RCTs, 175 participants.

*Nabilone 0.5 mg to 1 mg/day versus amitriptyline 10 to 20 mg, 2 weeks*

- Sleep quality (Insomnia Severity Index): MD 3.2; 95% CI 1.2 to 5.3; 1 EC, 31 participants.
- Restorative sleep (Leeds Sleep Evaluation Questionnaire): MD 0.5; 95% CI 0.00 to 1.0; 1 RCT, 31 participants.
- Alertness (Leeds Sleep Evaluation Questionnaire): MD 0.3; 95% CI -0.2 to 0.8; 1 RCT, 31 participants.

*Nabilone up to 2 mg/day versus codeine up to 240 mg/day, 6 weeks*

- Sleep pattern (different tools): MD 0.2; 95% CI -0.10 to 0.50; p = 0.20; 1 cross-over RCT, 71 participants.

*CBD (different doses, regimens, and duration) versus placebo*

- Sleep pattern (different tools): SMD -0.06; 95% CI -0.23 to 0.11; I² = 46%; 8 RCTs, 961 participants.

*Nabiximols (different doses, regimens, and duration) versus placebo*

- Sleep pattern (different tools): SMD -0.24; 95% CI -0.35 to -0.14; I² = 61%; 21 RCTs, 3,659 participants.

Tourette syndrome *(Curtis 2009 – high quality; Bilbao 2022; Oikonomou 2022 – critically low quality)*

*Oral dronabinol 10 mg/day versus placebo, 8 weeks*

- Tic severity: SMD -1.01, 95% CI -1.58 to -0.44; p = 0.0005; 1 RCT, 24 participants.

Tuberous sclerosis *(Oliveira 2025 – critically low quality)*

*CBD 25mg/kg/day versus placebo, 16 weeks*

- Reduction in number of seizures from baseline (% reduction): RR 29.1%; 95% CI 12.7 to 42.4; p < 0.01; 1 RCT, 151 participants.

*CBD 50mg/kg/day versus placebo, 16 weeks*

- Reduction in number of seizures from baseline: RD 28.4%; 95% CI 11.8 to 41.8; p = 0.02; 1 RCT, 148 participants.

Ulcerative colitis *(Kang 2025 – critically low quality; Kafil 2018a - high quality)*

*Cannabinoids (different dosages and presentations) versus placebo*

- Disease activity index (Lichtiger score): SMD -2.13; 95% CI -4.8 to -0.55; I2 = 90%; 2 RCTs, 51 participants.
- Endoscopic activity index (Mayo subscore): SMD -0.27; 95% CI -0.97 to 0.42; p = 0.43; I2 = 0%; 1 RCT, 102 participants.
- Quality of life (SF-36): SMD 1.79; 95% CI 0.23 to 3.34; I² = 77%; 2 RCTs, 51 participants.

*CBD + THC (up to 4.7%) 100 mg to 500 mg/day, oral capsules versus placebo, 10 weeks*

- Clinical remission (Mayo score < 2): CBD + THC may reduce the risk of remission (RR 0.94, 95% CI 0.39 to 2.25; 1 EC, 60 participants).
- Clinical response (reduction in Mayo score ≥ 3): CBD + THC may increase the likelihood of response (RR 1.37; 95% CI 0.59 to 3.21; 1 RCT, 60 participants).
- Quality of life (IBDQ): CBD + THC probably improves quality of life (SMD 17.4; 95% CI -3.45 to 38.25; 1 RCT, 53 participants).
- Serious adverse events: CBD + THC may reduce the risk of serious adverse events (RR 0.15; 95% CI 1.05 to 1.56; 1 RCT, 60 participants).
- Adverse events: CBD + THC probably increases the risk of adverse events (RR 1.28; 95% CI 0.01 to 2.83; 1 RCT, 60 participants).

*Cigarette containing 0.5 mg of cannabis, twice daily (up to 23 mg THC/day) versus placebo, 8 weeks*

- C-reactive protein (CRP, serum levels, mg/L): cannabis cigarettes may slightly reduce CRP levels (MD -0.3; 95% CI -1.35 to 0.75; 1 RCT, 28 participants).

Safety results presented by systematic reviews that exclusively evaluated adverse events as outcomes of interest.

Specific adverse events *(Souza 2022 - high quality)*

*CBD (300 mg/day to 1,000 mg/day or 20 mg/kg/day to 50 mg/kg/day) versus placebo, 1 to 16 weeks*

Gastrointestinal symptoms

- Increased appetite: 44 events (9.7%) in the CBD group versus 11 (3.8%) in the placebo group (p = 0.002; 12 EC, 745 participants).
- Increased TGO/TGP: 58 events (12.8%) in the CBD group versus 1 (0.3%) in the placebo group (p < 0.0001; 12 RCTs, 745 participants).
- Constipation: 14 events (3.1%) in the CBD group versus 6 (2.1%) in the placebo group (p > 0.05; 12 RCTs, 745 participants).
- Diarrhoea: 145 events (31.9%) in the CBD group versus 42 (14.4%) in the placebo group (p < 0.0001; 12 RCTs, 745 participants).
- Abdominal distension: 10 events (2.2%) in the CBD group versus 4 (1.4%) in the placebo group (p > 0.05; 12 RCTs, 745 participants).
- Abdominal pain: 20 events (4.4%) in the CBD group versus 4 (1.4%) in the placebo group (p = 0.031; 12 RCTs, 745 participants).
- Nausea: 46 events (10.1%) in the CBD group versus 22 (7.6%) in the placebo group (p > 0.05; 12 EC, 745 participants).
- Loss of appetite: 75 events (16.5%) in the CBD group versus 32 (11.0%) in the placebo group (p = 0.041; 12 RCTs, 745 participants).
- Any gastrointestinal symptoms: 270 events (59.5%) in the CBD group versus 89 (30.6%) in the placebo group (p < 0.0001; 12 RCTs, 745 participants).
- Vomiting: 35 events (7.7%) in the CBD group versus 11 (3.8%) in the placebo group (p = 0.029; 12 RCTs, 745 participants).

General symptoms

- Skin rash: 29 events (6.4%) in the CBD group versus 2 (0.7%) in the placebo group (p < 0.0001; 12 EC, 745 participants).
- Fatigue: 52 events (11.4%) in the CBD group versus 40 (13.8%) in the placebo group (p > 0.05; 12 RCTs, 745 participants).
- Fever: 11 events (2.4%) in the CBD group versus 21 (7.2%) in the placebo group (p = 0.002; 12 RCTs, 745 participants).
- Weight gain: 11 events (2.4%) in the CBD group versus 11 (3.8%) in the placebo group (p > 0.05; 12 RCTs, 745 participants).
- Lethargy: 26 events (5.7%) in the CBD group versus 15 (5.1%) in the placebo group (p > 0.05; 12 RCTs, 745 participants).
- Weight loss: 25 events (5.5%) in the CBD group versus 22 (7.5%) in the placebo group (p > 0.05; 12 RCTs, 745 participants).

Neurological symptoms

- Headache: 33 events (7.8%) in the CBD group versus 17 (5.8%) in the placebo group (p > 0.05; 12 RCTs, 745 participants).
- Headache: 33 events (7.8%) in the CBD group versus 17 (5.8%) in the placebo group (p > 0.05; 12 RCTs, 745 participants).
- Insomnia: 15 events (3.3%) in the CBD group versus 5 (1.7%) in the placebo group (p > 0.05; 12 RCTs, 745 participants).
- Drowsiness: 76 events (16.7%) in the CBD group versus 43 (14.8%) in the placebo group (p > 0.05; 12 RCTs, 745 participants).
- Dizziness: 6 events (1.3%) in the CBD group versus 0 (0.0%) in the placebo group (p > 0.05; 12 RCTs, 745 participants).

Respiratory symptoms

- Upper respiratory tract infection: 14 events (3.1%) in the CBD group versus 10 (3.4%) in the placebo group (p > 0.05; 12 EC, 745 participants).
- Nasopharyngitis: 24 events (5.3%) in the CBD group versus 12 (4.1%) in the placebo group (p > 0.05; 12 RCTs, 745 participants).

Serious adverse events *(Souza 2022 – high quality)*

*CBD 20 mg/kg/day to 50 mg/kg/day versus placebo, 2 to 16 weeks*

- Increase in TGO/TGP: 29 events (6.4%) in the CBD group versus 0 (0.0%) in the placebo group (p < 0.0001; 12 RCTs, 745 participants).
- Seizure: 6 events (1.3%) in the CBD group versus 1 (0.0%) in the placebo group (p > 0.05; 12 RCTs, 745 participants).
- Skin rash: 5 events (1.1%) in the CBD group versus 0 (0.0%) in the placebo group (p > 0.05; 12 RCTs, 745 participants).

Any adverse events *(Chhabra 2024 - critically low quality)*

*Cannabinoids* (different dosages and presentations) versus placebo*

- Proportion of participants with at least one adverse event: RR 1.09; 95% CI 1.02 to 1.16; I² = 54%; 12 RCTs, 1,949 participants.

**CBD 5 to 50 mg/kg/day or Nabiximols (CBD 30 mg/day + THC 32.4 mg/day) or enriched extract (CBD 420 mg/day + THC 21 mg/day; CBD 0.9 to 21 mg/day + 0.1 to 1.75 mg/day) or transdermal gel (250 mg/day to 500 mg/day) versus placebo, 3 to 29.5 weeks*

Treatment discontinuation due to adverse events *(Chhabra 202432 – critically low quality)*

*Cannabinoids* (different dosages and presentations) versus placebo*

- Proportion of participants who dropped out of the clinical trial: RR 1.28; 95% CI 0.99 to 1.67; I² = 34%; 16 RCTs, 2,538 participants.

**CBD (5 to 50 mg/kg/day or 200 to 800 mg/day) or Nabiximols (CBD 30 mg/day + THC 32.4 mg/day) or enriched extract (CBD 420 mg/day + THC 21 mg/day; CBD 0.9 to 21 mg/day + 0.1 to 1.75 mg/day) or nabilone (1 mg/day to 8 mg/day) or THC (10 mg/m²asc/day) versus placebo or active control, 3 to 29.5 weeks.*

*CBD: cannabidiol; CT: clinical trial; CI: confidence interval; I²: inconsistency test (statistical heterogeneity); m²bwa: square metres of body surface area; RR: relative risk; GOT: glutamic oxaloacetic transaminase; GPT: glutamic pyruvic transaminase; THC: Δ-9-tetrahydrocannabinol.*
