# Supplementary material for: Efficacy and Safety of Cannabis Derivates and Their Synthetic Analogs. Overview of Systematic Reviews
Source: Phytother Res. 2026 Jun 23;40(11):6112–29. doi: 10.1002/ptr.70403 (PMC13753713; doi:10.1002/ptr.70403)
Supplement: Supplementary file 2 [file PTR-40-6112-s002.docx]

Supplementary material 4

Complete assessment of the studies incluofd using the AMSTAR-2 tool.

| **ID** | **Item 1** | **Item 2** | **Item 3** | **Item 4** | **Item 5** | **Item 6** | **Item 7** | **Item 8** | **Item 9** | **Item 10** | **Item 11** | **Item 12** | **Item 13** | **Item 14** | **Item 15** | **Item 16** | **AMSTAR-2*** | | |
| --- | --- | --- | --- | --- | --- | --- | --- | --- | --- | --- | --- | --- | --- | --- | --- | --- | --- | --- | --- |
| Alofrman 2023 | Yes | No | No | No | Yes | No | No | Yes | No | No | NA | NA | Yes | No | NA | Yes | 5 of 13 | 38,46 | Critically low |
| Amato 2017 | Yes | No | No | Yes | Yes | No | Yes | Partially Yes | Yes | No | Yes | Yes | Yes | Yes | Yes | Yes | 11 of 16 | 68,75 | Low |
| Aminilari 2021 | Yes | Yes | No | Yes | Yes | Yes | No | Yes | Yes | Yes | Yes | Yes | Yes | Yes | Yes | Yes | 14 of 16 | 87,5 | Low |
| Andreae 2015 | Yes | Yes | No | No | No | Yes | No | Yes | Yes | No | Yes | Yes | Yes | No | Yes | Yes | 10 of 16 | 62,5 | Critically low |
| Aviram 2017 | Yes | No | No | No | No | No | No | Yes | No | No | Yes | No | Yes | Yes | No | Yes | 6 of 16 | 37,5 | Critically low |
| Bajtel 2022 | Yes | Yes | No | No | No | Yes | Yes | Yes | Yes | No | No | No | No | Yes | Yes | Yes | 9 of 16 | 56,25 | Critically low |
| Barakji 2023 | Yes | Yes | No | Yes | Yes | Yes | No | Yes | Yes | No | Yes | Yes | Yes | Yes | Yes | Yes | 13 of 16 | 81,25 | Low |
| Belgers 2023 | Yes | No | No | Yes | Yes | No | No | Yes | Yes | No | Yes | No | No | Yes | Yes | Yes | 7 of 16 | 43,75 | Critically low |
| Bilbao 2022 | Yes | Yes | No | No | No | Yes | Yes | Yes | Yes | No | Yes | No | Yes | No | No | Yes | 8 of 16 | 50 | Critically low |
| Boland 2020 | Yes | Yes | No | Yes | Yes | Yes | No | Yes | Yes | Ñão | No | No | No | No | Yes | Yes | 9 of 16 | 56,25 | Critically low |
| Bonaccorso 2019 | Yes | No | No | No | No | No | No | No | No | No | NA | NA | No | No | NA | Yes | 2 of 13 | 15,38 | Critically low |
| Boychuk 2015 | Yes | No | No | No | Yes | Yes | Yes | No | No | No | NA | NA | No | No | NA | Yes | 5 of 13 | 38,46 | Critically low |
| Campbell 2001 | No | No | No | No | Yes | No | Yes | No | No | No | NA | NA | No | No | NA | Yes | 3 of 13 | 23,07 | Critically low |
| Carofnas 2025 | Yes | No | No | No | Yes | Yes | No | No | Yes | No | NA | NA | No | Yes | NA | Yes | 6 of 13 | 46,15 | Critically low |
| Charernboon 2020 | Yes | No | No | No | No | Yes | Yes | Yes | Yes | No | NA | NA | Yes | Yes | NA | Yes | 8 of 13 | 61,53 | Critically low |
| Chesney 2020 | Yes | Yes | No | No | Yes | Yes | Yes | Yes | Yes | No | Yes | No | No | No | No | Yes | 9 of 16 | 56,25 | Critically low |
| Chhabra 2024 | Yes | Yes | No | Yes | Yes | Yes | No | Yes | Yes | No | Yes | No | No | Yes | Yes | Yes | 11 of 16 | 68,75 | Critically low |
| Choi 2024 | Yes | No | No | No | Yes | Yes | No | Yes | No | No | Yes | No | Yes | No | NA | Yes | 7 of 15 | 46,66 | Critically low |
| Chow 2020 | Yes | No | No | No | Yes | No | No | No | Yes | No | Yes | No | No | No | NA | Yes | 7 of 15 | 46,66 | Critically low |
| Chow 2025 | Yes | No | No | No | Yes | No | No | No | Yes | No | Yes | No | Yes | No | Yes | Yes | 7 of 16 | 43,75 | Critically low |
| Coelho 2024 | Yes | No | No | No | Yes | No | No | Yes | Yes | No | NA | NA | No | No | NA | Yes | 5 of 13 | 38,46 | Critically low |
| Curtis 2009 | Yes | Yes | No | Yes | Yes | Yes | Yes | Yes | Yes | Yes | Yes | Yes | Yes | Yes | Yes | Yes | 15 of 16 | 93,75 | High |
| Ofshpanof 2015 | Yes | No | Ñão | No | No | Yes | No | Yes | No | Yes | NA | NA | Yes | Yes | NA | Yes | 7 of 13 | 53,84 | Critically low |
| Fazlollahi 2023 | Yes | No | No | Yes | Yes | Yes | Yes | Yes | Yes | No | No | Yes | Yes | No | NA | Yes | 10 of 15 | 66,66 | Critically low |
| Fillipini 2022 | Yes | Yes | No | Yes | Yes | Yes | Yes | Yes | Yes | Yes | Yes | Yes | Yes | Yes | Yes | Yes | 15 of 16 | 93,75 | High |
| Fisher 2021 | Yes | Yes | No | Yes | Yes | Yes | Yes | Yes | Yes | No | Yes | Yes | Yes | Yes | Yes | Yes | 14 of 16 | 87,5 | High |
| Fitzcharles 2016a | Yes | No | No | No | Yes | Yes | Yes | Yes | Yes | Yes | NA | NA | Yes | Yes | NA | Yes | 10 of 13 | 76,9 | Critically low |
| Fitzcharles 2016b | Yes | No | No | Yes | Yes | Yes | Yes | No | Partially Yes | No | NA | NA | No | No | NA | No | 5 of 13 | 38,46 | Critically low |
| Gazendam 2020 | Yes | No | No | No | Yes | Yes | No | No | Yes | No | No | No | Yes | Yes | NA | Yes | 7 of 15 | 46,7 | Critically low |
| Gedin 2022 | Yes | Yes | No | No | Yes | Yes | Yes | Yes | Yes | Yes | Yes | No | No | No | No | Yes | 10 of 16 | 62,5 | Critically low |
| Gloss 2014 | Yes | Yes | No | Yes | Yes | Yes | Yes | Yes | Yes | Yes | NA | NA | Yes | Yes | NA | Yes | 12 of 13 | 92,3 | High |
| Guerrini 2024 | Yes | No | No | Yes | Yes | No | No | Yes | Yes | No | Yes | No | No | No | NA | Yes | 7 of 15 | 46,66 | Critically low |
| Hammond 2021 | Yes | No | No | Yes | Yes | No | No | Yes | Yes | No | Yes | No | Yes | No | NA | Yes | 8 of 15 | 53,3 | Critically low |
| Hassan 2018 | Yes | No | No | Partially Yes | No | No | No | No | Partially Yes | No | NA | NA | No | No | NA | No | 1 of 13 | 7,7 | Critically low |
| Hauser 2019 | Yes | Yes | No | Yes | Yes | Yes | Yes | Yes | Yes | Yes | Yes | Yes | Yes | Yes | Yes | Yes | 15 of 16 | 93,75 | High |
| Hauser 2023 | Yes | Yes | No | Yes | Yes | Yes | Yes | Yes | Yes | Yes | Yes | Yes | Yes | Yes | Yes | Yes | 16 of 16 | 100 | High |
| Iskedjian 2007 | Yes | No | No | Partially Yes | Yes | Yes | No | Yes | Partially Yes | No | Yes | Yes | No | Yes | No | Yes | 8 of 16 | 50 | Critically low |
| Jeddi 2024 | Yes | Yes | No | Yes | Yes | Yes | Yes | Yes | Yes | No | Yes | Yes | Yes | Yes | Yes | Yes | 14 of 16 | 87,5 | Moderate |
| Johal 2020 | Yes | No | No | Partially Yes | Yes | Yes | No | Yes | Yes | No | Yes | Yes | Yes | Yes | No | Yes | 10 of 16 | 62,5 | Low |
| Kafil 2018a | Yes | Yes | No | Yes | Yes | Yes | Yes | Yes | Yes | Yes | NA | NA | Yes | Yes | NA | Yes | 12 of 13 | 92,3 | High |
| Kafil 2018b | Yes | Yes | No | Yes | Yes | Yes | Yes | Yes | Yes | Yes | NA | NA | Yes | Yes | NA | Yes | 12 of 13 | 92,3 | High |
| Kang 2025 | Yes | Yes | No | No | Yes | Yes | No | Partially Yes | Yes | No | Yes | No | No | No | NA | Yes | 7 of 15 | 46,67 | Critically low |
| Kleiner 2023 | Yes | Yes | No | Partially Yes | Yes | Yes | No | No | Yes | No | Yes | No | No | No | NA | Yes | 7 of 14 | 50 | Critically low |
| Kock 2024 | Yes | Yes | No | No | Yes | Yes | No | Yes | Yes | No | Yes | No | No | Yes | NA | Yes | 9 of 15 | 60 | Critically low |
| Kuharic 2021 | Yes | Yes | No | Yes | Yes | Yes | Yes | Yes | Yes | Yes | Yes | Yes | Yes | Yes | NA | Yes | 14 of 15 | 93,33 | High |
| Kumar 2023 | Yes | Yes | No | No | Yes | Yes | No | No | Yes | No | Yes | No | No | No | NA | Yes | 7 of 15 | 46,67 | Critically low |
| Lakhan 2009 | Yes | No | No | No | No | Yes | No | Yes | Partially Yes | No | NA | NA | No | No | NA | Yes | 4 of 13 | 30,76 | Critically low |
| Lattanzi 2018a | Yes | No | No | No | Yes | Yes | No | Yes | Yes | No | No | No | No | No | NA | Yes | 6 of 15 | 40 | Critically low |
| Lattanzi 2018b | Yes | No | No | No | Yes | Yes | No | Yes | Yes | No | No | No | No | No | NA | Yes | 6 of 15 | 40 | Critically low |
| Lattanzi 2020 | Yes | No | No | Yes | Yes | Yes | No | Yes | Yes | Yes | Yes | No | No | Yes | No | Yes | 10 of 16 | 62,5 | Low |
| Lim 2017 | Yes | No | No | No | No | No | No | Yes | Yes | No | NA | NA | Yes | No | NA | Yes | 5 of 13 | 38,46 | Critically low |
| Linley 2022 | Yes | Yes | No | No | Yes | No | No | Yes | Yes | No | Yes | No | No | No | NA | Yes | 7 of 16 | 43,75 | Critically low |
| Longo 2021 | Yes | No | No | No | No | No | No | No | No | No | NA | NA | No | No | NA | No | 1 of 13 | 7,69 | Critically low |
| Lopes-Júnior 2023 | Yes | No | No | No | Yes | Yes | No | Yes | Yes | Yes | Yes | No | No | No | NA | Yes | 8 of 15 | 53,33 | Critically low |
| Lutge 2013 | Yes | Yes | No | Yes | Yes | Yes | Yes | Yes | Yes | Yes | NA | NA | Yes | Yes | NA | Yes | 12 of 13 | 92,3 | High |
| Lynch 2015 | Yes | No | No | Partially Yes | Yes | No | No | Yes | Yes | No | NA | NA | No | No | NA | Yes | 5 of 13 | 38,46 | Critically low |
| Machado-Rocha 2008 | Yes | No | No | No | No | No | Yes | No | No | No | Yes | No | No | No | No | Yes | 4 of 16 | 25 | Critically low |
| Martin 2023 | Yes | No | No | No | No | No | No | No | No | No | NA | NA | No | No | NA | Yes | 2 of 13 | 15,4 | Critically low |
| Martin-Sanchez 2009 | Yes | No | No | Yes | No | No | No | No | Partially Yes | No | No | No | No | No | No | Yes | 3 of 16 | 18,75 | Critically low |
| McKee 2021 | Yes | No | No | Yes | Yes | No | No | Yes | Yes | No | Yes | No | No | No | NA | Yes | 7 of 15 | 46,66 | Critically low |
| McLoughlin 2014 | Yes | Yes | No | Yes | Yes | Yes | Yes | Yes | Yes | Yes | NA | NA | Yes | Yes | NA | Yes | 12 of 13 | 92,3 | High |
| McParland 2022 | Yes | No | No | No | Yes | No | No | Yes | Yes | No | Yes | No | Yes | Yes | NA | Yes | 8 of 15 | 53,3 | Critically low |
| Meng 2017 | Yes | Yes | No | Yes | Yes | Yes | No | Yes | Yes | No | Yes | Yes | Yes | Yes | NA | Yes | 12 of 15 | 80 | Low |
| Mucke 2018a | Yes | No | No | Yes | Yes | Yes | Yes | No | Yes | No | No | No | Yes | No | NA | Yes | 8 of 15 | 53,3 | Critically low |
| Mucke 2018b | Yes | Yes | No | Yes | Yes | Yes | Yes | Yes | Yes | Yes | Yes | Yes | Yes | Yes | Yes | Yes | 15 of 16 | 93,75 | High |
| Oikonomou 2022 | No | No | No | No | No | No | No | Partially Yes | No | No | NA | NA | No | No | NA | Yes | 1 of 13 | 7,69 | Critically low |
| Oliveira 2025 | Yes | Yes | No | No | Yes | Yes | No | No | Yes | No | Yes | No | No | NA | NA | Yes | 7 of 15 | 46,67 | Critically low |
| Paunescu 2020 | Yes | No | No | Partially Yes | No | No | No | Yes | No | No | NA | NA | No | No | NA | No | 2 of 13 | 15,38 | Critically low |
| Petzke 2016 | Yes | No | No | No | Yes | Yes | Yes | Yes | Yes | Yes | Yes | No | Yes | Yes | Yes | Yes | 12 of 16 | 75 | Critically low |
| Quintero 2022 | Yes | No | No | No | Yes | Yes | No | No | Yes | No | NA | NA | No | NA | NA | Yes | 5 of 12 | 41,67 | Critically low |
| Rabgay 2019 | Yes | Yes | No | Yes | No | Yes | No | No | Yes | No | Yes | No | No | No | NA | Yes | 7 of 16 | 43,75 | Critically low |
| Razmovski-Naumovski 2022 | Yes | Partially Yes | No | No | Yes | No | No | Partially Yes | Yes | No | NA | NA | No | Yes | NA | Yes | 5 of 13 | 38,46 | Critically low |
| Reechaye 2024 | Yes | No | No | No | Yes | Yes | No | No | Yes | No | NA | NA | No | No | NA | Yes | 5 of 15 | 33,33 | Critically low |
| Rovare 2017 | Yes | No | No | Yes | Yes | Yes | No | Yes | Yes | No | Yes | Yes | No | No | Yes | Yes | 10 of 16 | 62,5 | Critically low |
| Ruthirakuhan 2019 | - | - | - | - | - | - | - | - | - | - | - | - | - | - | - | - | - | - | - |
| Sainsbury 2021 | Yes | No | No | Partially Yes | Yes | Yes | No | Partially Yes | Yes | No | No | No | No | No | NA | Yes | 5 of 15 | 33,33 | Critically low |
| Seneca 2014 | Yes | No | No | No | No | No | No | No | No | No | No | No | No | No | NA | No | 1 of 15 | 6,66 | Critically low |
| Smith 2015 | Yes | Yes | Yes | Yes | Yes | Yes | Yes | Yes | Yes | No | Yes | Yes | Yes | Yes | Yes | Yes | 15 of 16 | 93,75 | High |
| Souza 2022 | Yes | Yes | No | Partially Yes | Yes | Yes | No | Yes | Yes | No | NA | NA | Yes | No | NA | Yes | 8 of 13 | 61,53 | High |
| Spanagel 2021 | Yes | Yes | No | No | Yes | Yes | No | Partially Yes | Yes | No | Yes | No | No | Yes | No | Yes | 8 of 16 | 50 | Critically low |
| Stevens 2017 | Yes | Yes | No | Partially Yes | Yes | Yes | No | Partially Yes | Yes | No | NA | NA | Yes | Yes | NA | Yes | 8 of 13 | 61,53 | High |
| Talwar 2023 | Yes | No | No | Yes | Yes | Yes | No | Yes | Yes | Yes | Yes | Yes | Yes | Yes | NA | Yes | 12 of 15 | 80 | Critically low |
| Tateo 2016 | Yes | No | No | Partially Yes | No | No | No | Yes | Partially Yes | No | NA | NA | No | No | NA | No | 2 of 13 | 15,38 | Critically low |
| Torres 2015 | Yes | Yes | No | Partially Yes | No | No | No | Yes | Yes | No | Yes | Yes | Yes | No | Yes | No | 8 of 13 | 61,53 | Low |
| Torres-Moreno 2018 | Yes | Yes | No | Yes | Yes | No | Partially Yes | Yes | Yes | Yes | Yes | Yes | Yes | Yes | Yes | Yes | 13 of 16 | 81,25 | High |
| Tramer 2001 | Yes | No | No | Yes | No | No | Yes | Yes | Yes | No | Yes | Yes | Yes | Yes | Yes | Yes | 11 of 16 | 68,75 | Moderate |
| Treves 2021 | Yes | Yes | No | Yes | Yes | Yes | No | Yes | Yes | Yes | Yes | No | Yes | Yes | No | Yes | 12 of 16 | 75 | Critically low |
| Tsai 2022 | Yes | Yes | No | Partially Yes | Yes | Yes | No | Partially Yes | Yes | No | Yes | No | No | No | No | Yes | 7 of 16 | 43,75 | Critically low |
| Vaid 2025 | Yes | Yes | No | Partially Yes | Yes | Yes | No | Partially Yes | Partially Yes | No | No | No | No | No | NA | Yes | 5 of 15 | 33,33 | Critically low |
| van ofn Elsen 2014 | Yes | No | No | No | Yes | No | No | Yes | Yes | No | NA | NA | Yes | Yes | NA | Yes | 7 of 13 | 53,84 | Critically low |
| Velayudhan 2024 | Yes | No | Yes | Yes | Yes | Yes | No | Yes | Yes | No | No | No | No | No | No | Yes | 8 of 16 | 50 | Critically low |
| Volz 2016 | Yes | No | No | No | Yes | No | No | No | Yes | No | NA | NA | Yes | NA | NA | Yes | 5 of 13 | 38,46 | Critically low |
| Votrubec 2022 | Yes | Yes | No | Yes | Yes | Yes | Yes | Yes | Yes | Yes | NA | NA | No | Yes | NA | Yes | 11 of 13 | 84,61 | High |
| Vuilleumier 2022 | Yes | No | No | No | Yes | Yes | No | Yes | Yes | No | NA | NA | No | Yes | NA | Yes | 7 of 13 | 53,84 | Critically low |
| Walitt 2016 | Yes | No | No | Yes | Yes | No | No | Yes | Yes | Yes | NA | NA | Yes | Yes | NA | Yes | 9 of 13 | 69,23 | Low |
| Wang 2019 | Yes | No | No | Yes | No | Yes | No | Yes | Yes | No | Yes | Yes | Yes | Yes | Yes | Yes | 11 of 16 | 68,75 | Moderate |
| Wang 2021 | Yes | Yes | Yes | Yes | Yes | Yes | Yes | Yes | Partially Yes | Yes | Yes | Yes | Yes | Yes | Yes | Yes | 15 of 16 | 93,75 | High |
| Wang 2024 | Yes | No | No | Yes | Yes | Yes | No | Yes | No | No | No | Yes | Yes | No | Yes | Yes | 9 of 16 | 56,25 | Critically low |
| Werneck 2018 | Yes | No | No | Partially Yes | Yes | Yes | No | Yes | Yes | No | NA | NA | No | No | NA | Yes | 6 of 13 | 46,15 | Critically low |
| Whiting 2015 | Yes | Yes | No | Yes | Yes | No | Yes | Yes | Yes | No | Yes | Yes | Yes | Yes | No | Yes | 12 of 16 | 75 | Moderate |
| Wong 2020 | Yes | No | No | Partially Yes | Yes | No | No | Partially Yes | Partially Yes | No | Yes | Yes | Yes | Yes | Yes | Yes | 8 of 16 | 50 | Low |
| Zhang 2022 | Yes | No | No | No | Yes | No | No | Yes | Yes | No | No | No | Yes | Yes | NA | Yes | 7 of 15 | 46,66 | Critically low |

*Items in dark blue: items considered critical by the AMSTAR-2 tool; NA: not applicable (no meta-analyses were conducted).*

*** Guide for applying the AMSTAR-2 tool.**

| **Item** | **Description** | **Guidance for judgement** |
| --- | --- | --- |
| 1 | Did the research questions and inclusion criteria for the review include the components of PICO? | *Yes / No*  **Yes**:  ✓ Population ✓ Intervention ✓ Comparator group  ✓ Outcome  **No** |
| 2 | Did the report of the review contain an explicit statement that the review methods were established prior to the conduct of the review and did the report justify any significant deviations from the protocol? | *Yes / Partial yes / No*  **Partial yes -** ALL BELOW  ✓ research question(s) ✓ search strategy ✓ inclusion/exclusion criteria ✓ a risk of bias assessment  **Yes -** ALL ABOVE AND BELOW  ✓ be registered in databases such as PROSPERO or OSF ✓ a meta-analysis/synthesis plan, if appropriate ✓ a plan for investigating causes of heterogeneity  ✓ provide justifications for any deviations from the original protocol  **No** |
| 3 | Did the review authors explain their selection of the study designs for inclusion in the review? | *Yes / No*  **Yes** - the review should satisfy ONE of the following  ✓ explanation for including only RCTs OR ✓ explanation for including only NRSI OR ✓ explanation for including both RCTs and NRSI  **No** |
| 4 | Did the review authors use a comprehensive literature search strategy? | *Yes / Partial yes /No* **Partial yes -** ALL BELOW  ✓ searched at least 2 databases (relevant to research question) ✓ provided key word and/or search strategy  ✓ justified publication restrictions (e.g. language)  **Yes -** ALL ABOVE AND BELOW  ✓ searched the reference lists / bibliographies of included studies ✓ searched trial/study registries ✓ included/consulted content experts in the field  ✓ where relevant, searched for grey literature ✓ conducted search within 24 months of completion of the review  **No** |
| 5 | Did the review authors perform study selection in duplicate? | *Yes / No*  **Yes** – either ONE of the following  ✓ at least two reviewers independently agreed on selection of eligible studies and achieved consensus on which studies to include OR ✓ two reviewers selected a sample of eligible studies and achieved good agreement (at least 80 percent), with the remainder selected by one reviewer  **No** |
| 6 | Did the review authors perform data extraction in duplicate? | *Yes / No*  **Yes** - either ONE of the following ✓ at least two reviewers achieved consensus on which data to extract from included studies OR ✓ two reviewers extracted data from a sample of eligible studies and achieved good agreement (at least 80 percent), with the remainder extracted by one reviewer  **No** |
| 7 | Did the review authors provide a list of excluded studies and justify the exclusions? | *Yes / Partial yes /No* **Partial yes**  ✓ provided a list of all potentially relevant studies that were read in full-text form but excluded from the review  **Yes -** ALL ABOVE AND  ✓ Justified the exclusion from the review of each potentially relevant study  **No** |
| 8 | Did the review authors describe the included studies in adequate detail? | *Yes / Partial yes / No* **Partial yes**  ✓ there is a description of the population, interventions, comparators, outcomes, and study designs  **Yes** - ALL ABOVE AND  ✓ there is a detailed description of the population, detailed intervention (including doses), detailed comparators (including doses), study context, and follow-up time  **No** |
| 9 | Did the review authors use a satisfactory technique for assessing the risk of bias (RoB) in individual studies that were included in the review? | *Yes / Partial yes / No* **Partial yes** ✓ the risk of bias for allocation concealment and masking of patients and outcome assessors was assessed (not necessary for objective outcomes such as all-cause mortality) **Yes -** ALL ABOVE AND  ✓ the risk of bias related to randomization and selective reporting of outcomes was assessed **No** |
| 10 | Did the review authors report on the sources of funding for the studies included in the review? | *Yes / No*  **Yes -** AT LEAST ONE BELOW ✓ there is information about the sources of funding for the individual studies included OR ✓ there are reports that the authors of the review sought this information, but it was not provided by the authors of the studies.  **No** |
| 11 | If meta-analysis was performed did the review authors use appropriate methods for statistical combination of results? | *Yes / No / Not apply* (NA)  **Yes -** ALL BELOW ✓ the authors justified combining the data in a meta-analysis AND  ✓ they used an appropriate weighted technique to combine study results and adjusted for heterogeneity if present AND  ✓ investigated the causes of any heterogeneity **No**  **NA -** No meta-analysis conducted |
| 12 | If meta-analysis was performed, did the review authors assess the potential impact of RoB in individual studies on the results of the meta-analysis or other evidence synthesis? | *Yes / No / Not apply* (NA)  **Yes -** AT LEAST ONE BELOW  ✓ included only low risk of bias RCTs OR ✓ if the pooled estimate was based on RCTs and/or NRSI at variable RoB, the authors performed analyses to investigate possible impact of RoB on summary estimates of effect  **No** **NA** - No meta-analysis conducted |
| 13 | Did the review authors account for RoB in individual studies when interpreting/ discussing the results of the review? | *Yes / No / Not apply* (NA)  **Yes -** AT LEAST ONE BELOW  ✓ included only low risk of bias RCTs OR  ✓ if RCTs with moderate or high RoB, or NRSI were included the review provided a discussion of the likely impact of RoB on the results  **No** **NA** - No meta-analysis conducted |
| 14 | Did the review authors provide a satisfactory explanation for, and discussion of, any heterogeneity observed in the results of the review? | *Yes / No*  **Yes -** AT LEAST ONE BELOW ✓ there was no significant heterogeneity in the results OR  ✓ if heterogeneity was present the authors performed an investigation of sources of any heterogeneity in the results and discussed the impact of this on the results of the review  **No** |
| 15 | If they performed quantitative synthesis did the review authors carry out an adequate investigation of publication bias (small study bias) and discuss its likely impact on the results of the review? | *Yes / No / Not apply* (NA)  **Yes**  ✓ performed graphical or statistical tests for publication bias and discussed the likelihood and magnitude of impact of publication bias  **No**  **NA** - No meta-analysis conducted |
| 16 | Did the review authors report any potential sources of conflict of interest, including any funding they received for conducting the review? | *Yes / No* **Yes** - AT LEAST ONE BELOW ✓ the authors reported no competing interests OR ✓ the authors described their funding sources and how they managed potential conflicts of interest  **No** |
| **Score**  *first column: number of items fulfilled/number of possible items. Only “yes” is considered fulfilled; “NA” excluded (considered impossible item)  *second column: percentage of adequacy (%). Only “yes” is considered adequate  **Rating**  ✓ critically low: more than one critical flaw  ✓ low: one critical flaw  ✓ moderate: more than one non-critical flaw  ✓ high: none or one non-critical flaw | | |

*NRCT: Non-randomized clinical trial; RCT: Randomized clinical trial; NA: Not applicable*
