# Supplementary material for: Efficacy and Safety of Cannabis Derivates and Their Synthetic Analogs. Overview of Systematic Reviews
Source: Phytother Res. 2026 Jun 23;40(11):6112–29. doi: 10.1002/ptr.70403 (PMC13753713; doi:10.1002/ptr.70403)
Supplement: Supplementary file 3 [file PTR-40-6112-s003.docx]

Supplementary material 2

Studies excluded (after reading the full text) and reasons for exclusion, considering the eligibility criteria of this overview.

|  | **Author, year** | **Reasons for exclusion** |
| --- | --- | --- |
| 1 | Akinyemi 2023 | Included other study designs than RCT |
| 2 | Arias 2024 | No risk of bias assessment performed |
| 3 | Asthana 2021 | No risk of bias assessment performed |
| 4 | Azadvari 2024 | Included other study designs than RCT |
| 5 | Bahji 2016 | No risk of bias assessment performed |
| 6 | Bahji 2020a | Included other study designs than RCT |
| 7 | Bahji 2020b | Included other study designs than RCT |
| 8 | Bougea 2020 | Included other study designs than RCT |
| 9 | Butler 2015 | Included other study designs than RCT |
| 10 | CADH 2010 | No risk of bias assessment performed |
| 11 | Cahill 2007 | Previous version of the Cahil 2011 systematic review |
| 12 | Cawkwell 2021 | Included other study designs than RCT |
| 13 | Chico 2024 | Included other study designs than RCT |
| 14 | Chou 2024 | Included other study designs than RCT |
| 15 | Corey 2005 | It is not referred to as a systematic review |
| 16 | Darkowska-Serafimovska 2018 | No risk of bias assessment performed |
| 17 | Desmarais 2020 | Included other study designs than RCT |
| 18 | Dykukha 2022 | Included other study designs than RCT |
| 19 | Elliot 2018 | Included other study designs than RCT |
| 20 | Farooquia 2019 | Included other study designs than RCT |
| 21 | Fife 2015 | Included other study designs than RCT |
| 22 | First 2020 | Included other study designs than RCT |
| 23 | Fusar-Poli 2020 | Included other study designs than RCT |
| 24 | Giardina 2024 | Included other study designs than RCT |
| 25 | Giossi 2022 | Included other study designs than RCT |
| 26 | Grossman 2021 | Included other study designs than RCT |
| 27 | Guillouard 2020 | Included other study designs than RCT |
| 28 | Guillouard 2021 | Included other study designs than RCT |
| 29 | Hakami 2025 | Included other study designs than RCT |
| 30 | Han 2024 | Included other study designs than RCT |
| 31 | Hanganu 2022 | Included other study designs than RCT |
| 32 | Hill 2022 | No risk of bias assessment performed |
| 33 | Hjorthoj 2009 | Included other study designs than RCT |
| 34 | Jawed 2024 | No risk of bias assessment performed |
| 35 | Johnson 2021 | No risk of bias assessment performed |
| 36 | Kang 2024 | No risk of bias assessment performed |
| 37 | Kansagara 2017 | Included other study designs than RCT |
| 38 | Khurshid 2021 | Included other study designs than RCT |
| 39 | Kim 2024 | No risk of bias assessment performed |
| 40 | Kopelli 2020 | No risk of bias assessment performed |
| 41 | Koppel 2014 | Included other study designs than RCT |
| 42 | Krishnan 2009 | Previous version of the Kuharic 2021 systematic review |
| 43 | Kuhathasan 2019 | It is not referred to as a systematic review |
| 44 | Landrigan 2022 | Included other study designs than RCT |
| 45 | Liu 2023 | Included other study designs than RCT |
| 46 | Lynch 2011 | Previous version of the Lynch 2015 systematic review |
| 47 | Madeo 2023 | Included other study designs than RCT |
| 48 | Maldonado 2010 | It is not referred to as a systematic review |
| 49 | Martinez-Paz 2023 | Included other study designs than RCT |
| 50 | McParland 2021 | No risk of bias assessment performed |
| 51 | Mejia-Gomez 2021 | No risk of bias assessment performed |
| 52 | Morel 2021 | Meta-epidemiological study |
| 53 | Motaghi 2023 | Included other study designs than RCT |
| 54 | Mucke 2016 | Previous version of the Mucke 2018a systematic review |
| 55 | Narayan 2022 | Included other study designs than RCT |
| 56 | Nielsen 2019 | Included other study designs than RCT |
| 57 | Orsolini 2019 | Included other study designs than RCT |
| 58 | Parrella 2023 | No risk of bias assessment performed |
| 59 | Parovincaka 2023 | No risk of bias assessment performed. |
| 60 | Pathak 2020 | Included other study designs than RCT |
| 61 | Pavel 2021 | No risk of bias assessment performed |
| 62 | Peña 2018 | Included other study designs than RCT |
| 63 | Pinto 2022 | Included health participants |
| 64 | Pradhan 2018 | Included other study designs than RCT |
| 65 | Rathbone 2008 | Previous version of the McLoughlin 2014 systematic review |
| 66 | Redonnet 2025 | Included other study designs than RCT |
| 67 | Rodriguez-Almaraz 2020 | Included other study designs than RCT |
| 68 | Sagdeo 2022 | Narrative review |
| 69 | Santos 2020 | No risk of bias assessment performed |
| 70 | Sankaranarayanan 2018 | Included other study designs than RCT |
| 71 | Schaefer 2019 | Included other study designs than RCT |
| 72 | Senderovich 2023 | Included other study designs than RCT |
| 73 | Serafimovska 2020 | No risk of bias assessment performed |
| 74 | Sette 2023 | No risk of bias assessment performed |
| 75 | Sherpa 2022 | Included other study designs than RCT |
| 76 | Silvinato 2022 | Included other study designs than RCT |
| 77 | Suraev 2020 | Included other study designs than RCT |
| 78 | Tang 2022 | Included health participants |
| 79 | To 2023 | Included other study designs than RCT |
| 80 | Varshney 2023 | Included other study designs than RCT |
| 81 | Velayudhan 2021 | Previous version of the Velayudhan 2024 systematic review |
| 82 | Vinci 2022 | Included other study designs than RCT |
| 83 | Wiczek 2020 | Included other study designs than RCT |
| 84 | Williams 2022 | It is not referred to as a systematic review |
| 85 | Yang 2021 | Included other study designs than RCT |
| 86 | Youssef 2017 | Included other study designs than RCT |
| 87 | Zhang 2021 | Did not specifically assessed cannabis or cannabis derivates |

*RCT: randomsed controlled trial.*

**References**

Akinyemi E, Randhawa G, Longoria V, Zeine R. Medical marijuana effects in movement disorders, focus on huntington disease: a literature review. J Pharm Pharm Sci. 2020;23. doi: 10.18433/jpps30967

Arias AY, Vadell K, Vashist A, Kolishetti N, Lakshmana MK, Nair M, Liuzzi JP. Cannabidiol, a plant-derived compound, is an emerging strategy for treating cognitive impairments: comprehensive review of randomized trials. Front Pharmacol. 2024;15:1403147. doi: 10.3389/fphar.2024.1403147

Asthana N, Sewell D. A systematic review of the novel applications of cannabinoids in dementia care: food refusal and neuropsychiatric symptoms. Am J Geri Psych. 2021; 29(4):S60-1. doi: 10.1016/j.jagp.2021.01.052

Azadvari M, Pourshams M, Guitynavard F, Emami-Razavi SZ, Taftian-Banadkouki E, Ghajarzade M, Rastkar M. Cannabinoids for spasticity in patients with multiple sclerosis: a systematic review and meta-analysis. Mult Scler J Exp Transl Clin. 2024;10(4):20552173241282379. doi: 10.1177/20552173241282379

Bahji A, Meyyappan AC, Hawken ER. Cannabinoids for the neuropsychiatric symptoms of dementia: a systematic review and meta-analysis. Can J Psychiatry. 2020;65(6):365-376. doi: 10.1177/0706743719892717

Bahji A, Meyyappan AC, Hawken ER. Efficacy and acceptability of cannabinoids for anxiety disorders in adults: a systematic review & meta-analysis. J Psychiatr Res. 2020;129:257-264. doi: 10.1016/j.jpsychires.2020.07.030

Bahji A, Mazhar MN. Treatment of cannabis dependence with synthetic cannabinoids: a systematic review. Can J Addict. 2016;7(4):8-13.

Bougea A, Koros C, Simitsi AM, Chrysovitsanou C, Leonardos A, Stefanis L. Medical cannabis as an alternative therapeutics for Parkinsons' disease: systematic review. Complement Ther Clin Pract. 2020;39:101154. doi: 10.1016/j.ctcp.2020.101154

Butler M, Krebs E, Sunderlin B, Kane RL. Medical cannabis for non-cancer pain: a systematic review. Minnesota Evidence-based Practice Center, Minneapolis, Minnesota, EUA. 2015.

Cahill K, Ussher M. Cannabinoid type 1 receptor antagonists (rimonabant) for smoking cessation. Cochrane Database Syst Rev. 2007;(4):CD005353. doi: 10.1002/14651858.CD005353.pub3. Update in: Cochrane Database Syst Rev. 2011;(3):CD005353. doi: 10.1002/14651858.CD005353.pub4

Canadian Agency for Drug and Technologies in Health, CADTH. Cannabinoids for the management of neuropathic pain: review of clinical effectiveness. Health Tech Inq Service. 2010. Disponível em: <https://www.cda-amc.ca/sites/default/files/pdf/l0197_cannabinoids_neuropathic_pain_htis-2.pdf>

Cawkwell PB, Hong DS, Leikauf JE. Neurodevelopmental effects of cannabis use in adolescents and emerging adults with ADHD: a systematic review. Harv Rev Psychiatry. 2021;29(4):251-261. doi: 10.1097/HRP.0000000000000303

Chico SFV, Diaz DAM, Contreras-Puentes N. Use of cannabidiol in the treatment of drug-refractory epilepsy in children and young adults: a systematic review. J Neurosci Rural Pract. 2024;15(2):203-210. doi: 10.25259/JNRP_618_2023

Chou R, Ahmed AY, Bougatsos C, Morasco BJ, Gilbreath T, Dana T, Fu R. Living systematic review on cannabis and other plant-based treatments for chronic pain: 2023 update – Surveillance report 4. 2024 May. In: Living systematic review on cannabis and other plant-based treatments for chronic pain: interim progress and surveillance reports [Internet]. Rockville (MD): Agency for Healthcare Research and Quality (US). 2020.

Corey S. Recent developments in the therapeutic potential of cannabinoids. P R Health Sci J. 2005;24(1):19-26.

Darkovska-Serafimovska M, Serafimovska T, Arsova-Sarafinovska Z, Stefanoski S, Keskovski Z, Balkanov T. Pharmacotherapeutic considerations for use of cannabinoids to relieve pain in patients with malignant diseases. J Pain Res. 2018;11:837-842. doi: 10.2147/JPR.S160556

Desmarais A, Smiddy S, Reddy S, El-Dallal M, Erlich J, Feuerstein JD. Evidence supporting the benefits of marijuana for Crohn's disease and ulcerative colitis is extremely limited: a meta-analysis of the literature. Ann Gastroenterol. 2020;33(5):495-499. doi: 10.20524/aog.2020.0516

Dykukha I, Essner U, Schreiber H, Raithel LM, Penner IK. Effects of SativexⓇ on cognitive function in patients with multiple sclerosis: a systematic review and meta-analysis. Mult Scler Relat Disord. 2022;68:104173. doi: 10.1016/j.msard.2022.104173

Elliott J, DeJean D, Clifford T, Coyle D, Potter BK, Skidmore B, Alexander C, Repetski AE, Shukla V, McCoy B, Wells GA. Cannabis-based products for pediatric epilepsy: a systematic review. Epilepsia. 2019;60(1):6-19. doi: 10.1111/epi.14608

Farooqui MT, Khan MA, Cholankeril G, Khan Z, Mohammed Abdul MK, Li AA, Shah N, Wu L, Haq K, Solanki S, Kim D, Ahmed A. Marijuana is not associated with progression of hepatic fibrosis in liver disease: a systematic review and meta-analysis. Eur J Gastroenterol Hepatol. 2019;31(2):149-156. doi: 10.1097/MEG.0000000000001263

Fife TD, Moawad H, Moschonas C, Shepard K, Hammond N. Clinical perspectives on medical marijuana (cannabis) for neurologic disorders. Neurol Clin Pract. 2015;5(4):344-351. doi: 10.1212/CPJ.0000000000000162

First L, Douglas W, Habibi B, Singh JR, Sein MT. Cannabis use and low-back pain: a systematic review. Cannabis Cannabinoid Res. 2020;5(4):283-289. doi: 10.1089/can.2019.0077

Fusar-Poli L, Cavone V, Tinacci S, Concas I, Petralia A, Signorelli MS, Díaz-Caneja CM, Aguglia E. Cannabinoids for people with ASD: A systematic review of published and ongoing studies. Brain Sci. 2020;10(9):572. doi: 10.3390/brainsci10090572

Giardina A, Palmieri R, Ponticelli M, Antonelli C, Carlucci V, Colangelo M, Benedetto N, Di Fazio A, Milella L. Is a low dosage of medical cannabis effective for treating pain related to fibromyalgia? A pilot study and systematic review. J Clin Med. 2024;13(14):4088. doi: 10.3390/jcm13144088

Giossi R, Carrara F, Padroni M, Bilancio MC, Mazzari M, Enisci S, Romio MS, Boni G, Corrù F, Fittipaldo VA, Tramacere I, Pani A, Scaglione F, Fornasari D. Systematic review and meta-analysis seem to indicate that cannabinoids for chronic primary pain treatment have limited benefit. Pain Ther. 2022;11(4):1341-1358. doi: 10.1007/s40122-022-00434-5

Grossman S, Tan H, Gadiwalla Y. Cannabis and orofacial pain: a systematic review. Br J Oral Maxillofac Surg. 2022;60(5):e677-e690. doi: 10.1016/j.bjoms.2021.06.005

Guillouard M, Authier N, Pereira B, Soubrier M, Mathieu S. Cannabis use assessment and its impact on pain in rheumatic diseases: a systematic review and meta-analysis. Arthritis Rheum. 2020;72:2414-5. doi: 10.1002/art.41538

Guillouard M, Authier N, Pereira B, Soubrier M, Mathieu S. Cannabis use assessment and its impact on pain in rheumatologic diseases: a systematic review and meta-analysis. Rheumatology. 2021;60(2):549-556. doi: 10.1093/rheumatology/keaa534

Hakami AY, Alshehri FS. Therapeutic potential of cannabinoids in neurological conditions: a systematic review of clinical trials. Front Pharmacol. 2025;16:1521792. doi: 10.3389/fphar.2025.1521792

Hanganu B, Lazar DE, Manoilescu IS, Mocanu V, Butcovan D, Buhas CL, Szalontay AS, Ioan BG. Controversial link between cannabis and anticancer treatments-where are we and where are we going? A systematic review of the literature. Cancers. 2022;14(16):4057. doi: 10.3390/cancers14164057

Han K, Wang JY, Wang PY, Peng YC. Therapeutic potential of cannabidiol (CBD) in anxiety disorders: a systematic review and meta-analysis. Psychiatry Res. 2024;339:116049. doi: 10.1016/j.psychres.2024.116049

Hill KP, Gold MS, Nemeroff CB, McDonald W, Grzenda A, Widge AS, Rodriguez C, Kraguljac NV, Krystal JH, Carpenter LL. Risks and benefits of cannabis and cannabinoids in psychiatry. Am J Psychiatry. 2022;179(2):98-109. doi: 10.1176/appi.ajp.2021.21030320

Hjorthøj C, Fohlmann A, Nordentoft M. Reprint of Treatment of cannabis use disorders in people with schizophrenia spectrum disorders--a systematic review. Addict Behav. 2009;34(10):846-51. doi: 10.1016/j.addbeh.2009.02.003

Jawed B, Esposito JE, Pulcini R, Zakir SK, Botteghi M, Gaudio F, Savio D, Martinotti C, Martinotti S, Toniato E. The evolving role of cannabidiol-rich cannabis in people with autism spectrum disorder: a systematic review. Int J Mol Sci. 2024;25(22):12453. doi: 10.3390/ijms252212453

Johnson S, Ziegler J, August DA. Cannabinoid use for appetite stimulation and weight gain in cancer care: does recent evidence support an update of the European Society for Clinical Nutrition and Metabolism clinical guidelines? Nutr Clin Pract. 2021;36(4):793-807. doi: 10.1002/ncp.10639

Kang H, Schmoyer C, Weiss A, Lewis JD. Meta-anaysis of the therapeutic impact of cannabis and cannabinoid-derivatives in inflammatory bowel disease. Gastroenterol. 2024;166(5):S-812. doi: 10.1016/S0016-5085(24)02349-7

Kansagara D, O'Neil M, Nugent S, Freeman M, Low A, Kondo K, Elven C, Zakher B, Motu'apuaka M, Paynter R, Morasco BJ. Benefits and harms of cannabis in chronic pain or post-traumatic stress disorder: a systematic review [Internet]. Washington (DC): Department of Veterans Affairs (US). 2017.

Khurshid H, Qureshi IA, Jahan N, Went TR, Sultan W, Sapkota A, Alfonso M. A systematic review of fibromyalgia and recent advancements in treatment: is medicinal cannabis a new hope? Cureus. 2021;13(8):e17332. doi: 10.7759/cureus.17332

Kim Y, Min S, Jung JH, Park JH, Kim HL, Lee EK. Comparative effectiveness of fenfluramine and cannabidiol for dravet syndrome: a network meta-analysis. Value Health. 2024;27(12):S31. doi: 10.1016/j.jval.2024.10.161

Kopelli E, Samara M, Siargkas A, Goulas A, Papazisis G, Chourdakis M. The role of cannabidiol oil in schizophrenia treatment. A systematic review and meta-analysis. Clin Nutr ESPEN. 2020;40:P502. doi: 10.1016/j.clnesp.2020.09.287

Koppel BS, Brust JC, Fife T, Bronstein J, Youssof S, Gronseth G, Gloss D. Systematic review: efficacy and safety of medical marijuana in selected neurologic disorders: report of the Guideline Development Subcommittee of the American Academy of Neurology. Neurology. 2014;82(17):1556-63. doi: 10.1212/WNL.0000000000000363

Krishnan S, Cairns R, Howard R. Cannabinoids for the treatment of dementia. Cochrane Database Syst Rev. 2009;2009(2):CD007204. doi: 10.1002/14651858.CD007204.pub2

Kuhathasan N, Dufort A, MacKillop J, Gottschalk R, Minuzzi L, Frey BN. The use of cannabinoids for sleep: a critical review on clinical trials. Exp Clin Psychopharmacol. 2019;27(4):383-401. doi: 10.1037/pha0000285

Landrigan J, Bessenyei K, Leitner D, Yakovenko I, Fisk JD, Prentice JL. A systematic review of the effects of cannabis on cognition in people with multiple sclerosis. Mult Scler Relat Disord. 2022;57:103338. doi: 10.1016/j.msard.2021.103338

Liu S, He Z, Li J. Long-term efficacy and adverse effects of cannabidiol in adjuvant treatment of drug-resistant epilepsy: a systematic review and meta-analysis. Ther Adv Neurol Disord. 2023;16:17562864231207755. doi: 10.1177/17562864231207755

Lynch ME, Campbell F. Cannabinoids for treatment of chronic non-cancer pain: a systematic review of randomized trials. Br J Clin Pharmacol. 2011;72(5):735-44. doi: 10.1111/j.1365-2125.2011.03970.x

Madeo G, Kapoor A, Giorgetti R, Busardò FP, Carlier J. Update on cannabidiol clinical toxicity and adverse effects: a systematic review. Curr Neuropharmacol. 2023;21(11):2323-2342. doi: 10.2174/1570159X21666230322143401

Maldonado J, Álvarez Pinzón AM, Rodríguez Martínez M. Effectivity and side effects of central neuropathic pain treatment with cannabinoids in patients with multiple sclerosis. Rev Med. 2010;18(1):77-83.

Martinez-Paz C, García-Cabrera E, Vilches-Arenas Á. Effectiveness and safety of cannabinoids as an add-on therapy in the treatment of resistant spasticity in multiple sclerosis: a systematic review. Cannabis Cannabinoid Res. 2023;8(4):580-588. doi: 10.1089/can.2022.0254

Mücke M, Carter C, Cuhls H, Prüß M, Radbruch L, Häuser W. Cannabinoide in der palliativen versorgung: systematische übersicht und metaanalyse der wirksamkeit, verträglichkeit und sicherheit [Cannabinoids in palliative care: systematic review and meta-analysis of efficacy, tolerability and safety]. Schmerz. 2016;30(1):25-36. doi: 10.1007/s00482-015-0085-2

McParland A, Daniel K, Bhatia A, Clarke H, Aditya T, Brull R, Singh M. Cannabinoids and sleep health in patients with chronic neuropathic pain: a systematic review and meta-analysis. Sleep. 2021;44:A271-2. doi: 10.1093/sleep/zsab072.693

Mejia-Gomez J, Phung N, Philippopoulos E, Murphy K, Wolfman W. Effect of cannabis use in peri- and post-menopausal women: a systematic review. J Obstet Gynaecol Can. 2021;43(5):680-1. doi: 10.1016/j.jogc.2021.02.107

Morel A, Lebard P, Dereux A, Azuar J, Questel F, Bellivier F, Marie-Claire C, Fatséas M, Vorspan F, Bloch V. Clinical trials of cannabidiol for substance use disorders: outcome measures, surrogate endpoints, and biomarkers. Front Psychiatry. 2021;12:565617. doi: 10.3389/fpsyt.2021.565617

Motaghi E, Ghasemi-Pirbaluti M, Rashidi M, Alasvand M, Di Ciano P, Bozorgi H. The effect of tetrahydrocannabinol: cannabidiol oromucosal spray on cognition: a systematic review. Eur J Clin Pharmacol. 2023;79(3):371-381. doi: 10.1007/s00228-023-03454-y

Narayan AJ, Downey LA, Manning B, Hayley AC. Cannabinoid treatments for anxiety: a systematic review and consideration of the impact of sleep disturbance. Neurosci Biobehav Rev. 2022;143:104941. doi: 10.1016/j.neubiorev.2022.104941

Nielsen S, Murnion B, Campbell G, Young H, Hall W. Cannabinoids for the treatment of spasticity. Dev Med Child Neurol. 2019;61(6):631-638. doi: 10.1111/dmcn.14165

Orsolini L, Chiappini S, Volpe U, Berardis D, Latini R, Papanti GD, Corkery AJM. Use of medicinal cannabis and synthetic cannabinoids in post-traumatic stress disorder (PTSD): a systematic review. Medicina. 2019;55(9):525. doi: 10.3390/medicina55090525

Parovincaka J, Vella-Szijj J, Serracino-Inglott A. Medicinal cannabis use in rare diseases. Pharm Educ. 2023;23(3):254-5. doi: 10.46542/pe.2023.233.246258

Parrella NF, Hill AT, Enticott PG, Barhoun P, Bower IS, Ford TC. A systematic review of cannabidiol trials in neurodevelopmental disorders. Pharmacol Biochem Behav. 2023;230:173607. doi: 10.1016/j.pbb.2023.173607

Pathak S, Jeyabalan JB, Liu K, Cook P, Lange B, Kim S, Nadar R, Ward K, Watts Alexander CS, Kumar A, Dua K, Moore T, Govindasamy J, Dhanasekaran M. Assessing effects of cannabis on various neuropathologies: a systematic review. J Ayurveda Integr Med. 2024;15(3):100911. doi: 10.1016/j.jaim.2024.100911

Pavel A, Paun R, Valentin M. P.316 The use of cannabidiol in treating psychiatric disorder: a systematic review. Eur Neuropsychopharmacol. 2021;44(Supl 1):S50. doi: 10.1016/j.euroneuro.2021.01.077

Peña J, Jiménez C, Schmidt J. Do cannabinoids play a role in the control of glaucoma? Medwave. 2018;18(1):7144-e7144.

Pinto JS, Martel F. Effects of cannabidiol on appetite and body weight: a systematic review. Clin Drug Investig. 2022;42(11):909-919. doi: 10.1007/s40261-022-01205-y

Pradhan RR, Pradhan SR, Mandal S, Pradhan DR. A systematic review of marijuana use and outcomes in patients with myocardial infarction. Cureus. 2018;10(9):e3333. doi: 10.7759/cureus.3333

Rathbone J, Variend H, Mehta H. Cannabis and schizophrenia. Cochrane Database Syst Rev. 2008;(3):CD004837. doi: 10.1002/14651858.CD004837.pub2. Update in: Cochrane Database Syst Rev. 2014;(10):CD004837. doi: 10.1002/14651858.CD004837.pub3

Redonnet B, Eren F, Avenin G, Melchior M, Mary-Krause M. Efficacy of cannabidiol alone or in combination with Δ-9-tetrahydrocannabinol for the management of substance use disorders: an umbrella review of the evidence. Addiction. 2025;120(5):813-834. doi: 10.1111/add.16745

Rodriguez-Almaraz JE, Chang S, Clarke J, Oberheim-Bush NA, Taylor J, Buerki R, Berger M, Zablotska L, Lobach I, Butowski N. A systematic review and meta-analysis examining the effects of cannabis and its derivatives in adults with malignant CNS tumors. Neurooncol Pract. 2020;7(4):376-383. doi: 10.1093/nop/npaa013

Sagdeo A, Askari A, Ball P, Morrissey H. Exploring the efficacy and safety of cannabis in the management of fibromyalgia. Intern J Curr Pharm Res. 2022;14(1):27-30. doi: 10.22159/ijcpr.2022v14i1.44109

Sankaranarayanan A, Wilding H, Neill E, Castle D. A critical systematic review of evidence for cannabinoids in the treatment of schizophrenia. Psychiatric Annals. 2018;48(5):214-23. doi: 10.3928/00485713-20180409-01

Santos RG, Guimarães FS, Crippa JAS, Hallak JEC, Rossi GN, Rocha JM, Zuardi AW. Serious adverse effects of cannabidiol (CBD): a review of randomized controlled trials. Expert Opin Drug Metab Toxicol. 2020;16(6):517-526. doi: 10.1080/17425255.2020.1754793

Schaefer M, Tafelski S, Häuser W. Efficacy, tolerability, and safety of cannabinoids for chemotherapy-induced nausea and vomiting. Med Cannabis Cannabinoids. 2019;2(2):81. doi: 10.1159/000500623

Senderovich H, Wagman H, Zhang D, Vinoraj D, Waicus S. The effectiveness of cannabis and cannabis derivatives in treating lower back pain in the aged population: a systematic review. Gerontology. 2022;68(6):612-624. doi: 10.1159/000518269

Serafimovska T, Darkovska-Serafimovska M, Stefkov G, Arsova-Sarafinovska Z, Balkanov T. Pharmacotherapeutic considerations for use of cannabinoids to relieve symptoms of nausea and vomiting induced by chemotherapy. Folia Med. 2020;62(4):668-678. doi: 10.3897/folmed.62.e51478

Sette CVM, Schoueri JH, De Iracema Gomes Cubero D, Del Giglio A. Use of medical cannabis in cancer-related symptons: a systematic review. J Clin Oncol. 2023;41(16):e24170.

Sherpa ML, Shrestha N, Ojinna BT, Ravi N, Shantha Kumar V, Choday S, Parisapogu A, Tran HH, Kc A, Elshaikh AO. Efficacy and safety of medical marijuana in migraine headache: a systematic review. Cureus. 2022;14(12):e32622. doi: 10.7759/cureus.32622

Silvinato A, Floriano I, Bernardo WM. Use of cannabidiol in the treatment of epilepsy: Lennox-Gastaut syndrome, Dravet syndrome, and tuberous sclerosis complex. Rev Assoc Med Bras. 2022;68(10):1345-1357. doi: 10.1590/1806-9282.2022D689

Suraev AS, Marshall NS, Vandrey R, McCartney D, Benson MJ, McGregor IS, Grunstein RR, Hoyos CM. Cannabinoid therapies in the management of sleep disorders: a systematic review of preclinical and clinical studies. Sleep Med Rev. 2020;53:101339. doi: 10.1016/j.smrv.2020.101339

Tang Y, Tonkovich KL, Rudisill TM. The effectiveness and safety of cannabidiol in non-seizure-related indications: a systematic review of published randomized clinical trials. Pharmaceut Med. 2022;36(6):353-385. doi: 10.1007/s40290-022-00446-8

To J, Davis M, Sbrana A, Alderman B, Hui D, Mukhopadhyay S, Bouleuc C, Case AA, Amano K, Crawford GB, de Feo G, Tanco K, Garsed J. MASCC Guideline: cannabis for cancer-related pain and risk of harms and adverse events. Support Care Cancer. 2023;31(4):202. doi: 10.1007/s00520-023-07662-1. Erratum in: Support Care Cancer. 2023;31(6):323. doi: 10.1007/s00520-023-07789-1

Varshney K, Patel A, Ansari S, Shet P, Panag SS. Cannabinoids in treating Parkinson's disease symptoms: a systematic review of clinical studies. Cannabis Cannabinoid Res. 2023;8(5):716-730. doi: 10.1089/can.2023.0023

Velayudhan L, McGoohan K, Bhattacharyya S. Safety and tolerability of natural and synthetic cannabinoids in adults aged over 50 years: a systematic review and meta-analysis. PLoS Med. 2021;18(3):e1003524. doi: 10.1371/journal.pmed.1003524

Vinci A, Ingravalle F, Bardhi D, Cesaro N, Frassino S, Licata F, Valvano M. Cannabinoid therapeutic effects in inflammatory bowel diseases: a systematic review and meta-analysis of randomized controlled trials. Biomedicines. 2022;10(10):2439. doi: 10.3390/biomedicines10102439

Wiczek MC. The effectiveness of cannabidiol in rheumatic disease pain: a systematic review [Master’s alternative plan paper, Minnesota State University, Mankato]. Cornerstone: a collection of scholarly and creative works for Minnesota State University, Mankato. 2020. Disponível em: <https://cornerstone.lib.mnsu.edu/> etds/974/

Yang M, Zhang YL, Smith CM, Hou Y-N, Deng G, Mao JJ, Feng Y, Wang H. Herbal formula mazirenwan (hemp seed pill) for constipation: a systematic review with meta-analysis. J Altern Complement Med. 2021;27(11):19. doi: 10.1089/acm.2021.29097.abstracts

Youssef NA, Schneider MP, Mordasini L, Ineichen BV, Bachmann LM, Chartier-Kastler E, Panicker JN, Kessler TM. Cannabinoids for treating neurogenic lower urinary tract dysfunction in patients with multiple sclerosis: a systematic review and meta-analysis. BJU Int. 2017;119(4):515-521. doi: 10.1111/bju.13759

Zhang L, Wang J, Wang C. Efficacy and safety of antiseizure medication for Lennox-Gastaut syndrome: a systematic review and network meta-analysis. Dev Med Child Neurol. 2022;64(3):305-313. doi: 10.1111/dmcn.15072
