# Supplementary material for: Efficacy and Safety of Cannabis Derivates and Their Synthetic Analogs. Overview of Systematic Reviews
Source: Phytother Res. 2026 Jun 23;40(11):6112–29. doi: 10.1002/ptr.70403 (PMC13753713; doi:10.1002/ptr.70403)
Supplement: Supplementary file 4 [file PTR-40-6112-s004.docx]

Supplementary material 1

Search strategies for each database and search results obtained on 9 April 2025.

| **Database** | **Search strategy** | **Results** |
| --- | --- | --- |
| Cochrane Database of Systematic Reviews (CDSR) | #1 MeSH descriptor: [Cannabinoids] explode all trees  #2 MeSH descriptor: [Cannabidiol] explode all trees  #3 MeSH descriptor: [Cannabinol] explode all trees  #4 MeSH descriptor: [Dronabinol] explode all trees  #5 MeSH descriptor: [Cannabis] explode all trees  #6 MeSH descriptor: [Cannabaceae] explode all trees  #7 Cannabinoid OR Cannabidiol OR Epidiolex OR "1,3-Benzenediol, 2-(3-methyl-6-(1-methylethenyl)-2-cyclohexen-1-yl)-5-pentyl-, (1R-trans)-" OR Cannabinol OR Dronabinol OR "delta(9)-THC" OR "9-ene-Tetrahydrocannabinol" OR "9 ene Tetrahydrocannabinol" OR THC OR "delta(1)-Tetrahydrocannabinol" OR "delta(1)-THC" OR "delta(9)-Tetrahydrocannabinol" OR Tetrahydrocannabinol OR "Tetrahydrocannabinol, (6a-trans)-Isomer" OR "Tetrahydrocannabinol, Trans-Isomer" OR "Tetrahydrocannabinol, Trans Isomer" OR "Tetrahydrocannabinol, (6aS-cis)-Isomer" OR "Tetrahydrocannabinol, Trans-(+-)-Isomer" OR Marinol OR "Tetrahydrocannabinol, (6aR-cis)-Isomer" OR Cannabis OR Marijuana OR (Hemp Plant) OR (Hemp Plants) OR (Plant, Hemp) OR (Plants, Hemp) OR Marihuana OR (Cannabis indica) OR (Cannabis sativa) OR Hemp OR Hemps OR Hashish OR Hashishs OR Bhang OR Bhangs OR Ganja OR Ganjas OR Cannabaceae  #8 #1 OR #2 OR #3 OR #4 OR #5 OR #6 OR #7 | 191 |
| Embase | #1 'cannabinoid'/exp OR Cannabinoid  #2 'cannabidiol'/exp OR Cannabidiol OR Epidiolex OR '1,3-Benzenediol, 2-(3-methyl-6-(1-methylethenyl)-2-cyclohexen-1-yl)-5-pentyl-, (1R-trans)-'  #3 'cannabinol'/exp OR Cannabinol  #4 'dronabinol'/exp OR Dronabinol OR 'delta(9)-THC" OR "9-ene-Tetrahydrocannabinol' OR '9 ene Tetrahydrocannabinol' OR THC OR 'delta(1)-Tetrahydrocannabinol' OR 'delta(1)-THC' OR 'delta(9)-Tetrahydrocannabinol' OR Tetrahydrocannabinol OR 'Tetrahydrocannabinol, (6a-trans)-Isomer' OR 'Tetrahydrocannabinol, Trans-Isomer' OR 'Tetrahydrocannabinol, Trans Isomer' OR 'Tetrahydrocannabinol, (6aS-cis)-Isomer' OR 'Tetrahydrocannabinol, Trans-(+-)-Isomer' OR Marinol OR 'Tetrahydrocannabinol, (6aR-cis)-Isomer'  #5 'cannabis'/exp OR Cannabis OR Marijuana OR (Hemp Plant) OR (Hemp Plants) OR (Plant, Hemp) OR (Plants, Hemp) OR Marihuana OR (Cannabis indica) OR (Cannabis sativa) OR Hemp OR Hemps OR Hashish OR Hashishs OR Bhang OR Bhangs OR Ganja OR Ganjas  #6 'Cannabaceae'/exp OR Cannabaceae  #7 #1 OR #2 OR #3 OR #4 OR #5 OR #6  #8 'review'/exp  #9 (literature NEAR/3 review*):ti,ab  #10 'meta analysis'/exp  #11 'systematic review'/exp  #12 #8 OR #9 OR #10 OR #11  #13 medline:ti,ab OR medlars:ti,ab OR embase:ti,ab OR pubmed:ti,ab OR cinahl:ti,ab OR amed:ti,ab OR psychlit:ti,ab OR psyclit:ti,ab OR psychinfo:ti,ab OR psycinfo:ti,ab OR scisearch:ti,ab OR cochrane:ti,ab  #14 retracted AND article  #15 #13 OR #14  #16 #12 AND #15  #17 (systematic* NEAR/2 (review* OR overview)):ti,ab  #18 (meta*anal*:ti,ab OR meta:ti,ab) AND anal*:ti,ab OR 'meta anal*':ti,ab OR metaanal*:ti,ab OR metanal*:ti,ab  #19 #16 OR #17 OR #18  #20 #7 AND #19  #21 #20 AND [embase]/lim NOT ([embase]/lim AND [medline]/lim) | 1.553 |
| Epistemonikos | #1 Cannabinoids OR Cannabidiol OR Cannabinol OR Dronabinol OR Cannabis OR Cannabaceae  Filters:  Publication type: systematic review  PubMed Central: no PMC  Cochrane reviews: no | 1.420 |
| MEDLINE (PubMed) | #1 "Cannabinoids"[Mesh] OR Cannabinoid OR "Cannabidiol"[Mesh] OR Cannabidiol OR Epidiolex OR "1,3-Benzenediol, 2-(3-methyl-6-(1-methylethenyl)-2-cyclohexen-1-yl)-5-pentyl-, (1R-trans)-" OR "Cannabinol"[Mesh] OR Cannabinol OR "Dronabinol"[Mesh] OR Dronabinol OR "delta(9)-THC" OR "9-ene-Tetrahydrocannabinol" OR "9 ene Tetrahydrocannabinol" OR THC OR "delta(1)-Tetrahydrocannabinol" OR "delta(1)-THC" OR "delta(9)-Tetrahydrocannabinol" OR Tetrahydrocannabinol OR "Tetrahydrocannabinol, (6a-trans)-Isomer" OR "Tetrahydrocannabinol, Trans-Isomer" OR "Tetrahydrocannabinol, Trans Isomer" OR "Tetrahydrocannabinol, (6aS-cis)-Isomer" OR "Tetrahydrocannabinol, Trans-(+-)-Isomer" OR Marinol OR "Tetrahydrocannabinol, (6aR-cis)-Isomer" OR "Cannabis"[Mesh] OR Cannabis OR Marijuana OR (Hemp Plant) OR (Hemp Plants) OR (Plant, Hemp) OR (Plants, Hemp) OR Marihuana OR (Cannabis indica) OR (Cannabis sativa) OR Hemp OR Hemps OR Hashish OR Hashishs OR Bhang OR Bhangs OR Ganja OR Ganjas OR "Cannabaceae"[Mesh] OR Cannabaceae  #2 (((systematic review[ti] OR systematic literature review[ti] OR systematic scoping review[ti] OR systematic narrative review[ti] OR systematic qualitative review[ti] OR systematic evidence review[ti] OR systematic quantitative review[ti] OR systematic meta-review[ti] OR systematic critical review[ti] OR systematic mixed studies review[ti] OR systematic mapping review[ti] OR systematic cochrane review[ti] OR systematic search and review[ti] OR systematic integrative review[ti]) NOT comment[pt] NOT (protocol[ti] OR protocols[ti])) NOT MEDLINE [subset]) OR (Cochrane Database Syst Rev[ta] AND review[pt]) OR systematic review[pt]  #3 #1 AND #2 | 1.398 |
| **Total** | | 4.562 |
