# Supplementary material for: Efficacy and Safety of Cannabis Derivates and Their Synthetic Analogs. Overview of Systematic Reviews
Source: Phytother Res. 2026 Jun 23;40(11):6112–29. doi: 10.1002/ptr.70403 (PMC13753713; doi:10.1002/ptr.70403)
Supplement: Supplementary file 5 [file PTR-40-6112-s005.docx]

Supplementary material 3

Main characteristics of the included systematic reviews.

| **Author, year** | **Population** | **Intervention** | **Comparator** | **Aim of the review** | **Number RCTs** | **Search date** | **Funding source** | **Conflicts of interests** | **AMSTAR-2** | |
| --- | --- | --- | --- | --- | --- | --- | --- | --- | --- | --- |
| *Last name of first author, year of publication* | *Health condition* | *Name of cannabis derivative or synthetic analogue* | *Name of comparators* | *Effectiveness and/or safety* | *Number of RCTs included* | *Date on which the searches were conducted* | *Main/primary source of funding* | *Present/absent declaration. Description of conflicts* | *Score*  *(% of suitability)* | *Classification (high, moderate, low, critically low)* |
| **Alderman 2023** | Cancer related gastrointestinal symptoms | Cannabinoids | Placebo, active control | Effectiveness, safety | 31 | 12/11/2021 | Not reported | No conflict | 38.46 | Critically low |
| **Amato 2017** | Chronic neuropathic pain, multiple sclerosis, nausea and vomiting after chemotherapy | Cannabinoids | Placebo | Effectiveness, safety | 41 | 09/09/2016 | World Health Organisation | No conflict | 68.75 | Low |
| **Aminilari 2021** | Sleep disorders | Cannabinoids | Placebo, active control | Effectiveness, safety | 38 | 19/01/2021 | None declared | No conflict | 87.5 | Low |
| **Andreae 2015** | Chronic neuropathic pain | Inhaled cannabis | Placebo | Effectiveness, safety | 5 | 30/04/2014 | National Institutes of Health, United States | No conflict | 62.5 | Critically low |
| **Aviram 2017** | Chronic pain, post-operative pain | Cannabinoids | Placebo, active control | Effectiveness, safety | 43 | 31/07/2015 | None declared | No conflict | 37.5 | Critically low |
| **Bajtel 2022** | Any illness/condition | Dronabinol, nabilone | Placebo | Safety | 16 | 21/02/2020 | Ministry for Innovation and Technology, Hungary | No conflict | 56.25 | Critically low |
| **Barakji 2023** | Pain | Cannabinoids | Placebo | Effectiveness, safety | 65 | 01/10/2019 | Lykfeldt and Wife Foundation, Canada | No conflict | 81.25 | Low |
| **Belgers 2023** | Chronic diseases of the central nervous system, cancer | Cannabinoids | Placebo, active control | Effectiveness | 17 | 02/08/2021 | Anita Veldman Foundation, Netherlands | One author declared receiving fees from pharmaceutical companies. | 43.75 | Critically low |
| **Bilbao 2022** | Any illness/condition | Cannabidiol, dronabinol, nabilone, nabiximols | Placebo, active control | Effectiveness, safety | 152 | 31/10/2021 | Bundesministerium für Bildung und Forschung, Germany | No conflict | 50 | Critically low |
| **Boland 2020** | Cancer pain | Cannabinoids | Placebo, active control | Effectiveness, safety | 6 | 01/08/2019 | None declared | No conflict | 56.25 | Critically low |
| **Bonaccorso 2019** | Psychiatric disorders | Cannabidiol | Placebo | Effectiveness, safety | 27 | 31/01/2017 | Not reported | No conflict | 15.38 | Critically low |
| **Boychuk 2015** | Chronic non-cancer neuropathic pain | Cannabinoids | Placebo, active control | Effectiveness, safety | 13 | 30/04/2013 | Not reported | No conflict | 38.46 | Critically low |
| **Campbell 2001** | Pain | Cannabinoids | Placebo, active control | Effectiveness, safety | 9 | 31/10/1999 | Swiss National Research Foundation, Switzerland | One author declared that he was an employee of Pfizer. | 23.07 | Critically low |
| **Cardenas 2025** | Post-operative pain | Cannabinoids | Placebo, conventional analgesics | Effectiveness, safety | 5 | 22/11/2022 | Faculty of Medicine of the Universidad El Bosque, Colombia | Not reported | 46.15 | Critically low |
| **Charernboon 2020** | Dementia | Cannabinoids | Placebo, active control | Effectiveness, safety | 5 | 30/06/2019 | Not reported | No conflict | 61.53 | Critically low |
| **Chesney 2020** | Any illness/condition | Cannabidiol | Placebo | Safety | 13 | 31/01/2019 | Not reported | No conflict | 56.25 | Critically low |
| **Chhabra 2024** | Any illness/condition  Age <18 years old | Cannabinoids | Placebo, active control | Safety | 23 | 01/03/2024 | Canadian Institutes of Health Research Cannabis Team, Canada | No conflict | 68.75 | Critically low |
| **Choi 2024** | Neuropathic pain, peripheral neuropathy | Cannabinoids | Placebo | Effectiveness, safety | 14 | 30/11/2022 | Not reported | No conflict | 46.66 | Critically low |
| **Chow 2020** | Post-chemotherapy nausea and vomiting | Cannabinoids orais | Placebo, active control | Effectiveness, safety | 24 | 31/12/2018 | Not reported | No conflict | 46.66 | Critically low |
| **Chow 2025** | Post-chemotherapy nausea and vomiting | Cannabinoids | Placebo, active control | Effectiveness, safety | 26 | 31/03/2024 | Not reported | No conflict | 43.75 | Critically low |
| **Coelho 2024** | Anxiety | Cannabidiol | Placebo | Effectiveness, safety | 11 | 31/12/2023 | Fundação de Amparo à Pesquisa do Estado de Goiás (FAPEG), Brazil | No conflict | 38.46 | Critically low |
| **Curtis 2009** | Tourette syndrome | Cannabinoids | Placebo, active control | Effectiveness, safety | 2 | 31/10/2008 | Birmingham and Solihull Mental Health Trust, United Kingdon | No conflict | 93.75 | High |
| **Deshpande 2015** | Chronic non-cancer pain | Cannabinoids non-synthetic inhalants or cigarettes | Placebo, active control | Effectiveness, safety | 6 | 30/04/2014 | Not reported | No conflict | 53.84 | Critically low |
| **Fazlollahi 2023** | Epilepsy | Cannabidiol | Placebo | Safety | 9 | 04/08/2022 | Shahid Beheshti University of Medical Sciences, Iran | One author declared receiving fees from the Brain Mapping Research Centre | 66.66 | Critically low |
| **Filippini 2022** | Multiple sclerosis | Cannabinoids | Placebo, active control | Effectiveness, safety | 25 | 31/12/2021 | Not reported | No conflict | 93.75 | High |
| **Fisher 2021** | Pain | Cannabinoids | Placebo, active control | Effectiveness, safety | 36 | 30/04/2019 | Not reported | All authors declare that they receive fees from research centres and/or pharmaceutical laboratories | 87.5 | High |
| **Fitzcharles 2016a** | Rheumatic pain | Cannabinoids | Placebo, active control | Effectiveness, safety | 2 | 30/04/2015 | Not reported | All authors declare that they receive fees from research centres and/or pharmaceutical laboratories. | 76.9 | Critically low |
| **Fitzcharles 2016b** | Rheumatic diseases | Cannabinoids | Placebo, active control | Effectiveness, safety | 4 | 31/01/2015 | Not reported | Not reported | 38.46 | Critically low |
| **Gazendam 2020** | Acute pain | Cannabinoids | Placebo | Effectiveness, safety | 6 | 21/01/2019 | None declared | No conflict | 46.7 | Critically low |
| **Gedin 2022** | Pain | Cannabinoids | Placebo | Effectiveness, safety | 20 | 30/09/2021 | Not reported | No conflict | 62.5 | Critically low |
| **Gloss 2014** | Epilepsy | Cannabinoids | Placebo, active control | Effectiveness, safety | 4 | 09/09/2013 | Not reported | No conflict | 92.3 | High |
| **Guerrini 2024** | Dravet syndrome | Cannabidiol | Placebo, active control | Effectiveness, safety | 8 | 12/12/2022 | Biocodex SA, France | All authors declare that they receive fees from research centres and/or pharmaceutical laboratories | 46.66 | Critically low |
| **Hammond 2021** | Cachexia | Cannabinoids | Placebo, active control | Effectiveness, safety | 5 | 28/02/2020 | None declared | No conflict | 53.3 | Critically low |
| **Hassan 2018** | Chronic pain | Cannabinoids | Placebo, active control | Effectiveness, safety | 21 | 15/03/2017 | Not reported | Not reported | 7.7 | Critically low |
| **Hauser 2019** | Dor oncológica | Cannabinoids | Placebo, active control | Effectiveness, safety | 5 | 31/12/2018 | None declared | No conflict | 93.75 | High |
| **Hauser 2023** | Dor oncológica | Cannabinoids | Placebo, active control | Effectiveness, safety | 14 | 26/01/2023 | National Institute for Health Research, United Kingdon | No conflict | 100 | High |
| **Iskedjian 2007** | Multiple sclerosis | Cannabinoids | Placebo | Effectiveness, safety | 7 | 30/06/2006 | Bayer Inc., Toronto, Canada | Not reported | 50 | Critically low |
| **Jeddi 2024** | Chronic non-cancer pain | Cannabinoids | Opioids | Effectiveness, safety | 90 | 31/03/2021 | Canadian Institutes of Health Research, Canada | No conflict | 87.5 | Moderate |
| **Johal 2020** | Chronic non-cancer pain | Cannabinoids | Placebo | Effectiveness, safety | 36 | 15/12/2028 | None declared | No conflict | 62.5 | Low |
| **Kafil 2018a** | Retocolite ulcerativa | Cannabinoids | Placebo, active control | Effectiveness, safety | 2 | 02/01/2018 | Crohn's and Colitis Canada, Canada | No conflict | 92.3 | High |
| **Kafil 2018b** | Doença de Crohn | Cannabinoids | Placebo, active control | Effectiveness, safety | 3 | 17/10/2018 | Crohn's and Colitis Canada, Canada | One author declared receiving fees from pharmaceutical companies | 92.3 | High |
| **Kang 2025** | Doença inflamatória intestinal | Cannabinoids | Placebo, active control | Effectiveness, safety | 8 | 31/07/2023 | Not reported | All authors declare that they receive fees from research centres and/or pharmaceutical laboratories. | 46.67 | Critically low |
| **Kleiner 2023** | Multiple sclerosis | Nabiximols | Placebo | Effectiveness, safety | 9 | 15/10/2021 | None declared | No conflict | 50 | Critically low |
| **Kock 2024** | Psychiatric disorders | Cannabinoids | Placebo | Effectiveness, safety | 9 | 30/06/2024 | None declared | No conflict | 60 | Critically low |
| **Kuharic 2021** | Demência | Cannabinoids | Placebo, active control | Effectiveness, safety | 4 | 08/07/2021 | National Institute for Health Research, United Kingdon | No conflict | 93.33 | High |
| **Kumar 2023** | Retocolite ulcerativa | Cannabinoids | Placebo | Effectiveness, safety | 4 | 31/07/2023 | Not reported | No conflict | 46.67 | Critically low |
| **Lakhan 2009** | Epilepsy | Cannabidiol/THC | Placebo | Effectiveness, safety | 6 | 30/04/2009 | Not reported | No conflict | 30.76 | Critically low |
| **Lattanzi 2018a** | Síndrome de Lennox-Gastaut | Cannabidiol | Placebo | Effectiveness, safety | 2 | 21/05/2018 | None declared | All authors declare that they receive fees from research centres and/or pharmaceutical laboratories | 40.00 | Critically low |
| **Lattanzi 2018b** | Epilepsy | Cannabidiol | Placebo | Effectiveness, safety | 4 | 28/06/2018 | None declared | All authors declare that they receive fees from research centres and/or pharmaceutical laboratories | 40.00 | Critically low |
| **Lattanzi 2020** | Dravet syndrome | Cannabidiol | Placebo | Effectiveness, safety | 3 | 21/10/2019 | None declared | Four authors declared that they had received funding from different pharmaceutical companies as speakers, consultants, scholarship holders, or had been members of boards of directors or advisory boards | 62.5 | Low |
| **Lim 2017** | Psychiatric disorders, desordens do movimento, doenças neurodegenerativas | Cannabinoids | Placebo, active control | Effectiveness, safety | 24 | 30/04/2017 | Singapore Ministry of Health’s National Medical Research Council sob o Centre Grant Programme, Singapore | Not reported | 38.46 | Critically low |
| **Linley 2022** | Dravet syndrome | Cannabidiol | Fenfluramine | Effectiveness, safety | 5 | 30/11/2021 | Zogenix International, United Kingdon | The authors are employed by the manufacturing industry | 43.75 | Critically low |
| **Longo 2021** | Chronic pain | Cannabinoids | Placebo, active control | Effectiveness, safety | 13 | 21/11/2019 | Not reported | Not reported | 7.69 | Critically low |
| **Lopes-Júnior 2023** | Chronic pain | Cannabinoids | Placebo | Effectiveness, safety | 4 | 06/107/2023 | None declared | No conflict | 53.33 | Critically low |
| **Lutge 2013** | HIV/AIDS | Cannabinoids | Placebo, active control | Effectiveness, safety | 7 | 03/07/2012 | Cochrane HIV/AIDS Mentoring Programme,  South Africa | No conflict | 92.3 | High |
| **Lynch 2015** | Chronic non-cancer pain | Cannabinoids | Placebo, active control | Effectiveness, safety | 11 | 31/10/2014 | Not reported | One author declared a direct relationship with companies that develop cannabis. One author received a grant for a clinical trial of cannabis | 38.46 | Critically low |
| **Machado-Rocha 2008** | Post-chemotherapy nausea and vomiting | Cannabinoids | Placebo, active control | Effectiveness, safety | 30 | 31/12/2006 | None declared | No conflict | 25 | Critically low |
| **Martin 2023** | Psychotic disorders | Cannabidiol | Placebo, active control | Effectiveness, safety | 10 | 25/06/2022 | None declared | No conflict | 15.4 | Critically low |
| **Martin-Sanchez 2009** | Chronic pain | Cannabinoids | Placebo, active control | Effectiveness, safety | 18 | 28/02/2008 | Spain National Drug Plan, Spain | No conflict | 18.75 | Critically low |
| **McKee 2021** | Psychiatric disorders | Cannabinoids | Placebo, active control | Effectiveness, safety | 31 | 30/09/2020 | None declared | All authors received fees from pharmaceutical companies | 46.66 | Critically low |
| **McLoughlin 2014** | Schizophrenia | Cannabinoids | Placebo, active control | Effectiveness, safety | 8 | 12/08/2013 | University of Nottingham, United Kingdon | No conflict | 92.3 | High |
| **McParland 2022** | Chronic neuropathic pain | Cannabinoids | Placebo, active control | Effectiveness, safety | 8 | 26/03/2021 | University of Toronto, Canada | No conflict | 53.3 | Critically low |
| **Meng 2017** | Chronic neuropathic pain | Cannabinoids | Placebo, active control | Effectiveness, safety | 11 | 11/03/2016 | Department of Anesthesia and Pain Management at Toronto Western Hospital, Canada | No conflict | 80 | Low |
| **Mucke 2018a** | Cancer cachexia and HIV/AIDS | Cannabinoids | Placebo, active control | Effectiveness, safety | 9 | 15/03/2017 | Três autores receberam financiamento do Governo de Nova Gales do Sul, Australia | No conflict | 53.3 | Critically low |
| **Mucke 2018b** | Chronic neuropathic pain | Cannabinoids | Placebo, active control | Effectiveness, safety | 16 | 30/11/2017 | National Institute for Health Research, United Kingdon | One author declared receiving fees from pharmaceutical companies | 93.75 | High |
| **Oikonomou 2022** | Movement disorders | Cannabinoids | Placebo, active control | Effectiveness, safety | 7 | 31/03/2022 | Not reported | No conflict | 7.69 | Critically low |
| **Oliveira 2025** | Epilepsy | Cannabidiol | Placebo | Effectiveness, safety | 6 | 31/01/2024 | None declared | No conflict | 46.67 | Critically low |
| **Paunescu 2020** | Alzheimer's disease | Cannabinoids | Placebo | Effectiveness, safety | 6 | 31/03/2019 | Not reported | No conflict | 15.38 | Critically low |
| **Petzke 2016** | Chronic neuropathic pain | Cannabinoids | Placebo, active control | Effectiveness, safety | 15 | 30/11/2015 | Not reported | No conflict | 75 | Critically low |
| **Quintero 2022** | Chronic neuropathic pain | Cannabinoids | Placebo | Effectiveness, safety | 1 | 04/04/2022 | Universidad de La Sabana, Colombia | No conflict | 41.67 | Critically low |
| **Rabgay 2019** | Pain | Cannabinoids | Placebo, active control | Effectiveness, safety | 25 | 30/06/2017 | Naresuan Universit,  Thailand | No conflict | 43.75 | Critically low |
| **Razmovski-Naumovski 2022** | Cancer-related loss of appetite | Cannabinoids | Placebo, active control | Effectiveness, safety | 5 | 28/02/2021 | None declared | No conflict | 38.46 | Critically low |
| **Reechaye 2024** | Chronic neuropathic pain | Cannabinoids | Placebo | Effectiveness, safety | 5 | 31/08/2024 | None declared | No conflict | 33.33 | Critically low |
| **Rovare 2017** | Spasticity (multiple sclerosis, paraplegia) | Cannabinoids | Placebo, active control | Effectiveness, safety | 6 | 20/03/2017 | Conselho Nacional de Desenvolvimento Científico e Tecnológico (CNPq), Brazil | No conflict | 62.5 | Critically low |
| **Ruthirakuhan 2019*** | Alzheimer's disease | Cannabinoids | Placebo | Effectiveness, safety | 6 | 31/08/2018 | - | - | - | - |
| **Sainsbury 2021** | Chronic neuropathic pain | Cannabinoids | Placebo | Effectiveness, safety | 17 | 02/01/2021 | None declared | No conflict | 33.33 | Critically low |
| **Seneca 2014** | Chronic pain | Cannabinoids | Placebo | Effectiveness, safety | 6 | 15/02/2013 | None declared | No conflict | 6.66 | Critically low |
| **Smith 2015** | Post-chemotherapy nausea and vomiting | Cannabinoids | Placebo, active control | Effectiveness, safety | 23 | 31/01/2015 | Cochrane Gynaecological Cancer Review Group, United Kingdon | No conflict | 93.75 | High |
| **Souza 2022** | Any illness/condition | Cannabidiol | Placebo, active control | Safety | 12 | 31/05/2022 | None declared | One author declared having conducted studies for Janssen-Cilag, Torrent Pharm, Prati-Donaduzzi, Mantecorp, ArtMed, PurMed Global, and BSPG Pharm in the last three years. Three authors are co-inventors of two patents for cannabinoid compounds | 61.53 | High |
| **Spanagel 2021** | Any illness/condition | Cannabidiol, dronabinol, nabilone | Placebo, active control | Safety | 29 | 31/05/2021 | Bundesministerium für Bildung und Forschung e Deutsche Forschungsgemeinschaft, Germany | No conflict | 53.33 | Critically low |
| **Stevens 2017** | Acute pain | Cannabinoids | Placebo, active control | Effectiveness, safety | 4 | 20/08/2016 | None declared | No conflict | 61.53 | High |
| **Talwar 2023** | Epilepsy | Cannabidiol | Placebo | Effectiveness, safety | 6 | 01/06/2022 | National Institutes of Health, Departamento de Defesa e Texas A&M University Presidential X-Grant Award from Texas A&M University - United States | One author declared receiving grants from Incyte, Novartis, Gilead, and Astellas outside of the study | 80.0 | Critically low |
| **Tateo 2016** | Cancer pain | Cannabinoids | Placebo, active control | Effectiveness, safety | 8 | Not reported | Not reported | Not reported | 15.38 | Critically low |
| **Torres 2015** | Multiple sclerosis | Cannabinoids | Placebo, active control | Effectiveness, safety | 15 | 14/05/2015 | Not reported | Not reported | 61.53 | Low |
| **Torres-Moreno 2018** | Multiple sclerosis | Cannabinoids | Placebo | Effectiveness | 17 | 26/07/2016 | Ministerio de Sanidad, Servicios Sociales e Igualdad, Spain | No conflict | 81.25 | High |
| **Tramer 2001** | Post-chemotherapy nausea and vomiting | Cannabinoids | Placebo, active control | Effectiveness, safety | 30 | 31/08/2000 | Swiss National Science Foundation.Royal College of Nursing Institute RAE, Switzerland | No conflict | 68.75 | Moderate |
| **Treves 2021** | Any illness/condition Idade <=18 anos | Cannabinoids | Placebo, active control | Effectiveness, safety | 8 | 11/05/2020 | David R. Bloom Center for Pharmacy, Israel | One of the authors reported being a member of the safety monitoring committee for GW Research Ltd. studies. Two authors reported receiving grants from Canndoc for another study | 75 | Critically low |
| **Tsai 2022** | Spinal cord injuries | Cannabinoids | Placebo | Effectiveness, safety | 5 | 25/12/2021 | None declared | No conflict | 43.75 | Critically low |
| **Vaid 2025** | Crohn's disease | Cannabinoids | Placebo, active control | Effectiveness, safety |  | 28/02/2024 | Not reported | No conflict | 33.33 | Critically low |
| **van den Elsen 2014** | Any illness/condition Age > = 65 years old | Cannabinoids | Placebo, active control | Effectiveness, safety | 5 | 07/10/2013 | European Regional Development Fund (ERDF) | Not reported | 53.84 | Critically low |
| **Velayudhan 2024** | Any condition  Age >50 years old | Cannabinoids | Placebo, active control | Safety | 58 | 12/06/2023 | Parkinson’s UK, Psychiatry Research Trust, Rosetrees Trust, Alzheimer’s Research UK e National Institute of Health Research, United Kingdon | Two authors collaborated with studies by Beckley Canopy Therapeutics/Canopy Growth | 50 | Critically low |
| **Volz 2016** | Gastrointestinal diseases | Cannabinoids | Placebo | Effectiveness, safety | 1 | 31/03/2015 | None declared | One author received funding from Deutsche Morbus. Two authors have conducted studies for different pharmaceutical companies | 38.46 | Critically low |
| **Votrubec 2022** | D Orofacial pain | Cannabinoids | Placebo, active control | Effectiveness, safety | 5 | 11/07/2021 | None declared | No conflict | 84.61 | High |
| **Vuilleumier 2022** | Cannabis withdrawal | Cannabinoids | Placebo | Effectiveness, safety | 6 | 06/11/2021 | Open Access Publication Fund of the University of Duisburg-Essen, Germany | No conflict | 53.84 | Critically low |
| **Walitt 2016** | Fibromyalgia | Cannabinoids | Placebo, active control | Effectiveness, safety | 2 | 30/04/2016 | None declared | Two authors declared that they had received fees from different pharmaceutical companies | 69.23 | Low |
| **Wang 2019** | Cancer-related cachexia | Cannabinoids | Placebo | Effectiveness, safety | 3 | 31/12/2017 | Natural Science Foundation of China, China | No conflict | 68.75 | Moderate |
| **Wang 2021** | Chronic pain | Cannabinoids | Placebo | Effectiveness, safety | 32 | 07/02/2020 | Canadian Institutes of Health Research, Canada; n Australian National Health and Medical Research Council, Australia | Two authors received funding from different pharmaceutical companies. One author is the medical director of Canopy Growth Corporation | 93.75 | High |
| **Wang 2024** | Epilepsy | Cannabidiol | Placebo, active control | Effectiveness, safety | 6 | 03/01/2023 | None declared | No conflict | 56.25 | Critically low |
| **Werneck 2018** | Cannabis withdrawal | Cannabinoids | Placebo, active control | Effectiveness, safety | 10 | 01/09/2017 | None declared | One author declared that he had received fees from Pfizer to support smoking cessation training in Brazil | 46.15 | Critically low |
| **Whiting 2015** | Any illness/condition | Cannabinoids | Placebo, active control | Effectiveness, safety | 79 | 30/04/2015 | Swiss Federal Office of Public Health, Switzerland | The lead author reported that part of her time spent on this review was supported by a grant from the National Institute for Health Research (NIHR) | 75 | Moderate |
| **Wong 2020** | Chronic non-cancer pain | Cannabinoids | Placebo, active control | Effectiveness, safety | 43 | 31/12/2018 | Department of Anaesthesiology, Li Ka Shing Faculty of Medicine, The University of Hong Kong, Hong Kong | No conflict | 50 | Low |
| **Zhang 2022** | Schizophrenia | Cannabidiol | Placebo | Effectiveness, safety | 9 | 01/07/2020 | None declared | No conflict | 46.6 | Critically low |

**review not available in full text. CNPq: National Council for Scientific and Technological Development; ERDF: European Regional Development Fund; FOPH: Swiss Federal Office of Public Health; NIHR: National Institute for Health Research.*
